# Neuroplasticity of the extended amygdala in opioid withdrawal and prolonged opioid abstinence

**DOI:** 10.3389/fphar.2023.1253736

**Published:** 2023-11-16

**Authors:** Gary B. Kaplan, Benjamin L. Thompson

**Affiliations:** ^1^ Mental Health Service, VA Boston Healthcare System, Boston, MA, United States; ^2^ Department of Psychiatry, Boston University Chobanian & Avedisian School of Medicine, Boston, MA, United States; ^3^ Department of Pharmacology and Experimental Therapeutics, Boston University Chobanian & Avedisian School of Medicine, Boston, MA, United States; ^4^ Department of Psychiatry, Harvard Medical School, Boston, MA, United States

**Keywords:** opioid withdrawal, neuroplasticity, extended amygdala, opioid use disorder, morphine, heroin, addiction

## Abstract

Opioid use disorder is characterized by excessive use of opioids, inability to control its use, a withdrawal syndrome upon discontinuation of opioids, and long-term likelihood of relapse. The behavioral stages of opioid addiction correspond with affective experiences that characterize the opponent process view of motivation. In this framework, active involvement is accompanied by positive affective experiences which gives rise to “reward craving,” whereas the opponent process, abstinence, is associated with the negative affective experiences that produce “relief craving.” Relief craving develops along with a hypersensitization to the negatively reinforcing aspects of withdrawal during abstinence from opioids. These negative affective experiences are hypothesized to stem from neuroadaptations to a network of affective processing called the “extended amygdala.” This negative valence network includes the three core structures of the central nucleus of the amygdala (CeA), the bed nucleus of the stria terminalis (BNST), and the nucleus accumbens shell (NAc shell), in addition to major inputs from the basolateral amygdala (BLA). To better understand the major components of this system, we have reviewed their functions, inputs and outputs, along with the associated neural plasticity in animal models of opioid withdrawal. These models demonstrate the somatic, motivational, affective, and learning related models of opioid withdrawal and abstinence. Neuroadaptations in these stress and motivational systems are accompanied by negative affective and aversive experiences that commonly give rise to relapse. CeA neuroplasticity accounts for many of the aversive and fear-related effects of opioid withdrawal via glutamatergic plasticity and changes to corticotrophin-releasing factor (CRF)-containing neurons. Neuroadaptations in BNST pre-and post-synaptic GABA-containing neurons, as well as their noradrenergic modulation, may be responsible for a variety of aversive affective experiences and maladaptive behaviors. Opioid withdrawal yields a hypodopaminergic and amotivational state and results in neuroadaptive increases in excitability of the NAc shell, both of which are associated with increased vulnerability to relapse. Finally, BLA transmission to hippocampal and cortical regions impacts the perception of conditioned aversive effects of opioid withdrawal by higher executive systems. The prevention or reversal of these varied neuroadaptations in the extended amygdala during opioid withdrawal could lead to promising new interventions for this life-threatening condition.

## 1 Introduction

Opioid use disorder (OUD) is a major, enduring public health issue in the United States. The National Survey on Drug Use and Health reported that approximately 1.6 million individuals in the US met the diagnostic criteria for OUD in 2020 (SAMHSA, 2021). According to the fifth edition of the *Diagnostic and Statistical Manual of Mental Disorders fifth ed.* of the American Psychiatric Association (APA, 2013), OUD is characterized by impaired inhibitory control over involvement with opioids, role dysfunction due to recurrent opioid use, opioid use in physically hazardous situations, tolerance to the effects of opioids, physical dependence, a withdrawal syndrome upon discontinuation of opioids, and long-term likelihood of relapse, often due to persistent craving to use opioids and despite negative consequences (Schuckit, 2016).

Physical dependence in addiction has been used to describe the adaptations that result in withdrawal symptoms when opioids are discontinued. Opioid-induced physical dependence along with opioid withdrawal both produce morphological alterations at the cellular level, along with corresponding organizational changes among neural circuits that are associated with addiction-related behavioral adaptations (Thompson et al., 2021), so too is chronic opioid *withdrawal* associated with critical changes in neurophysiology, dendritic connectivity and molecular changes in key brain areas that make individuals more susceptible to developing OUD. The somatic symptoms that accompany abstinence from chronic opioid exposure and characterize the subjective phenomenon of withdrawal include vomiting, diarrhea, chills, muscle cramps and spasms, and tremor whereas the negative affective experiences include anxiety, dysphoria, insomnia, and heightened sensitivity to pain and stress (Kaplan et al., 2011). There is evidence that the underlying neurobiological vulnerability for OUD consists, in part, of opioid-induced structural and functional synaptic adaptations at the level of the stress system. Importantly, these neurophysiological changes to the stress system are accompanied by negative affective experiences that commonly give rise to relapse, even after prolonged periods of abstinence (Koob, 2020). A constellation of brain regions within the stress system, known as the “extended amygdala,” functions critically in the facilitation of the stress response to both external and internal stimuli by modulating the release and transmission of stress hormones, such as corticotrophin-releasing factor (CRF), and activating the hypothalamic-pituitary-adrenal (HPA) axis (Koob and Schulkin, 2019; Koob, 2020), as well as stress-related neurotransmitters, and the noradrenergic system.

*Our objective in this review is to summarize and synthesize the literature related to neuroadaptations within the extended amygdala during the withdrawal and abstinence phases of opioid addiction.* Because most of the neurobiological evidence related to opioid withdrawal comes from research using nonhuman animal models, and the diagnostic criteria for OUD within the Diagnostic and Statistical Manual of Mental Disorders fifth ed. comprises many items that are limited to human-related consequences of pathological opioid use (American Psychiatric Association, 2013), we use the term “opioid addiction” to refer to the trans-species phenomenon, unless otherwise specified. We begin our review by situating the behavioral and affective elements of opioid addiction within the allostatic framework proposed by Koob and colleagues who have provided a central role in its definition (Koob and Le Moal, 1997; Edwards and Koob, 2010; Shurman et al., 2010; Volkow and Koob, 2015; George and Koob, 2017; Koob and Schulkin, 2019). Here, we focus primarily on the relevance of withdrawal-relief craving as a motive force perpetuating pathological opioid involvement, and we describe how the different manifestations of craving correspond with the opponent process view of motivation, as well as how the physiological phenomenon of allostasis drives this process. Next, we describe how opioid-related allostasis disproportionately recruits the stress system in general and neurocircuits that are part of the extended amygdala in particular. We then summarize research related to the structural and functional plasticity that occur within the brain regions constituting the extended amygdala, specifically during the process of opioid withdrawal and prolonged abstinence. We conclude by considering the possible implications of the findings for the development of novel clinical interventions. Our main objective is to begin to elucidate some of the neurobiological underpinnings of OUD by providing examples of the relevant scientific findings with the hope that meaningful advances in etiology and prevention of OUD.

## 2 Theoretical framework for opioid addiction

There are many biological, genetic and environmental risk factors relevant to the development of OUD. Genetic risk factors for OUD include polymorphisms in several genes, including opioid receptor genes that have been associated with OUD (Kreek et al., 2012). Additionally, increased drug availability through prescription practices is important to the development of prescription opioid abuse (Volkow et al., 2011). In this last paper, Volkow and associates demonstrated that increases in opioid prescriptions were associated with increases in abuse and overdoses and the authors challenged existing opioid prescribing practices. Human studies have also reported a positive association between adverse life events and chronic distress. In animal models, stress exposure increases drug self-administration and reinstates drug seeking in drug-experienced animals suggesting stress-induced vulnerability to relapse (Sinha, 2013). Other studies have highlighted demographic factors of sex, race, and education to be important in risk for OUD (Bonar et al., 2020). For example, in prescription opioid abuse, females without college education were at greater risk than males without college. Furthermore, the risk of prescription opioid abuse was much higher for non-Hispanic whites while it is negligible for Hispanics (McCabe et al., 2017). In summary, genetics, white race, gender, lower educational and socioeconomic status, and chronic stress are all risk factors in the development of OUD.

One theoretical model that describes the development and persistence of opioid addiction within the allostatic framework suggested by the neuroscientist of addiction, George Koob and others (Koob and Le Moal, 1997; Edwards and Koob, 2010; Shurman et al., 2010; Volkow and Koob, 2015; George and Koob, 2017; Koob and Schulkin, 2019). We begin by discussing the relevance of addictive craving to the loss of control feature that defines the addiction cycle, prior to considering how the behavioral and affective correlates of the addiction cycle can be understood in terms of opponent process theory. We then explain what the concept of allostasis adds to our understanding of the negative experiences characterizing withdrawal and abstinence.

### 2.1 Craving and the loss of control

The defining feature of OUD, which distinguishes it (and all substance use disorders) as a clinical phenomenon from mere dependence, is the loss of inhibitory control (i.e., agency or autonomy) over involvement with opioids, despite negative consequences. For those who suffer with OUD, the thought of the almost certain consequences, whether personal, interpersonal, professional, that will accompany continued use, either do not come to mind or they are easily displaced and discounted in favor of the hedonic value associated with involvement (Bickel and Marsch, 2001). The pathological motivation to use opioids, despite the likely consequences, can be understood in terms of craving, which has been defined as an intense, urgent, and abnormal longing or yearning for involvement with a substance or activity (Anton, 1999; Sinha, 2013). From a phenomenological perspective, craving can be viewed as the experience whereby individuals with a substance use disorder are essentially pulled in the direction of values they do not find rationally valuable, or rather, by “wants they do not want to want” (Frankfurt, 1988). The paradox of this dilemma is that, although these addictive cravings are categorically out of control, they nevertheless represent a physiological strategy to regain control by stabilizing the internal milieu.

The type of craving characteristic of process (a) is referred to as “reward craving,” whereas the craving typical of an opposing process (b) is known as “withdrawal relief craving” (Heinz et al., 2003). When an individual’s intention is to control involvement [process (a)] through *moderation*, but reward craving becomes operative through an involuntary narrowing of their “salience landscape” (Hovhannisyan and Vervaeke, 2022), they report not being able to “stop, once they start.” On the other hand, when the intention is to control involvement through *abstinence* [process (b)], and the persistent acute discomfort of withdrawal becomes a salient motivator, affected individuals commonly report being driven by a desire for relief and, hence, not being able to “stop from starting again,” despite the negative consequences associated with previous involvement. Notably, the relief commonly sought by affected individuals typically emerges in the context of uncontrollable stress, which may be either exogenous (e.g., traumatic experiences, economic hardship, and social isolation) (Morgan et al., 2002; Laudet and White, 2008) or endogenous (e.g., the development of allostatic load in response chronic involvement with opioid) (Koob et al., 2014). While this depiction characterizes the loss of inhibitory control in terms of opponent process theory, it does not provide a mechanistic account for why control gets lost in the first place, nor why the out-of-control behavior not only persists but tends to worsen over time.

### 2.2 What allostasis adds

To address this gap in understanding, Koob and colleagues have adapted an allostatic model of physiological and affective regulation (Koob, 2004; 2009; Koob and Le Moal, 1997; 2008b; Solomon, 1980; Solomon and Corbit, 1974). Originally, allostasis was proposed as the innate tendency of organisms to contend with perceived or anticipated chronic stress (Sterling and Eyer, 1988; Schulkin, 2011) Importantly, the concept of allostasis is understood in distinction to the idea of homeostasis, which refers to a dynamic state of equilibrium or balance that has been hypothesized to underlie motivated behaviors (Ramsay and Woods, 2014). Whereas the homeostatic view of behavior, physiological and affective regulation or stability are achieved through counterbalancing processes, as in opponent process theory (Solomon, 1980), allostasis refers to the tendency of a system to self-regulate/stabilize through change or adaptation (Ramsay and Woods, 2014). The concept of homeostasis as applied to organismic motivation was derived from physics-based observations of inorganic matter and was adapted to explain the body’s use of feedback systems to self-stabilize in the presence of both internal and external perturbations. What the homeostatic framework essentially failed to provide, however, was an explanation for how an organism achieves self-stability less by real-time feedback and more by predictions about future experiences based on prior feedback, which are stored as a type of memory at the cellular level across multiple systems (Goldstein and Kopin, 2007, p. 116). *As such, an allostatic state can be defined as a chronic deviation of the regulatory system from its normal (homeostatic) operating level* (Koob, 2004)*, which can be either adaptive or maladaptive, depending upon the lesson learned and predictions made.*

In the case of opioid use, the hedonic effects that accompany involvement are mediated by sensor-effector loops within the reward system (Solomon and Corbit, 1974; Solomon, 1980; Koob and Le Moal, 1997; Koob, 2004; 2009; Koob and Le Moal, 2008), which, in turn, reflexively trigger sensor-effectors of the so-called anti-reward system that offset the excessive reward system activation (Koob and Le Moal, 2004; Koob, 2009; Koob and Schulkin, 2019). On this view, to compensate for the chronic involvement with reward via exposure to opioids, the anti-reward system becomes functionally “upregulated,” whereas the reward system is functionally “downregulated” (George and Koob, 2017). In addition, the stress system also becomes upregulated as it is conditioned to anticipate the aversive affective state that characterizes the withdrawal process through abstinence (Koob and Le Moal, 2008; Koob, 2020). What emerges is a chronically high state of endogenous stress and a blunted homeostatic “set point” (Koob and Le Moal, 1997; Ahmed and Koob, 1998).

Paradoxically, with addiction, inhibitory control (i.e., intentional self-regulation) is undermined by the body’s innate allostatic tendency to self-regulate through self-change. Notably, the affective changes that characterize opioid-induced allostasis supervene upon corresponding distinctive neuroadaptations, particularly in the extended amygdala. Such adaptations are neuroplastic events that include the restructuring of neuronal connections, electrophysiological changes within regions and circuits, and a host of complex neurotransmitter, neuromodulator, signaling and molecular changes and corresponding behavioral changes.

## 3 Experimental models of opioid abstinence and withdrawal

In the preceding section, we described how a new homeostatic set point emerges through allostasis in response to chronic stress, in addition to how chronic exposure to opioids upregulate the stress system both during the involvement stage and the abstinence/withdrawal stage. In this section, we briefly describe the experimental models commonly employed to investigate the phenomenon of withdrawal from opioids.

### 3.1 Models of abstinence and withdrawal from opioids

Withdrawal from opioids can be modeled in nonhuman animals, such as with mice implanted with morphine pellets or mini-osmotic pumps with opioids, which upon drug discontinuation produce significant increases in the somatic signs of opioid withdrawal, relative to vehicle pellet- or pump-implanted mice. In this model or by giving opioids repeatedly and at escalated doses or repeated self-administration (Harris and Aston-Jones, 1993), after administration of naloxone or naltrexone both of which are opioid antagonists, somatic withdrawal signs can be induced. In this non-contingent withdrawal model using morphine pellets, Kaplan and coworkers (1998; 1996) showed that somatic signs of withdrawal included jumping, wet-dog shakes, forepaw tremors, weight loss, and diarrhea. Both these negative affective and somatic states have been shown to accompany drug-induced neuroadaptations to the glutamate (GLU), dopamine (DA), norepinephrine (NE), and corticotrophin-releasing factor (CRF) systems, and they are believed to negatively reinforce abstinence, which in turn motivates reinstatement of opioid involvement via relief craving (Koob and Le Moal, 2005; George and Koob, 2017).

Another recent model of opioid withdrawal revealed that heroin-treated mice demonstrated both hyperalgesia and somatic signs (Alvarez-Bagnarol et al., 2022). Following behavioral assessments of hyperalgesia and somatic signs of withdrawal, this study examined neural activity using activity marker c-Fos expression. Using principal component analyses, they demonstrated that hyperalgesia was associated with c-Fos expression in the lateral hypothalamus, CeA, VTA, parabrachial nucleus, dorsal raphe, and LC. Somatic withdrawal was associated with c-Fos expression in the paraventricular nucleus of the thalamus, lateral habenula, dorsal raphe, and LC. In summary, hyperalgesia during spontaneous opioid withdrawal and somatic withdrawal resulted in c-Fos expression in autonomic and limbic brain regions.

#### 3.1.1 Opioid conditioned place aversion

An experimental measure of the negative affective and somatic experiences of withdrawal is derived from performance on a variation of the conditioned place preference (CPP) paradigm, known as conditioned place aversion (CPA). This technique involves training animals to associate the rewarding effects of opioid administration with a specific cue or environment/context, followed by the association of a different environment with the absence of the drug or place aversion. One study by Hand and others (1988) used a CPA approach to investigate the aversive effects of naloxone in morphine-implanted rats. In the study, rats that had received several days of morphine pellet implantation were confined to the naloxone-paired compartment of a CPP box immediately following the administration of naloxone. The rats were then allowed to freely explore their two-compartment apparatus, and the time spent in each compartment was recorded. The difference between time spent in the naloxone-paired compartment post-conditioning, minus the time spent in the same compartment pre-conditioning, represented the naloxone-induced change in preference. Place aversion, which is indicated by a negative score, is a behavioral proxy for the negative experiences during withdrawal (Hand et al., 1988; Koob et al., 2014).

#### 3.1.2 Suppression of operant conditioning

Another paradigm used to study opioid withdrawal employs a variation of operant conditioning, whereby the animals learn to press a lever or engage in a specific response to receive an intravenous infusion of the drug (Bouton, 2019). Once self-administration (SA) behavior is established, the suppression of operant conditioning begins. During the suppression phase, animals are subjected to a procedure to induce opioid withdrawal symptoms, which can involve the administration of an opioid receptor antagonist, such as naloxone which precipitates withdrawal, or a cessation of drug availability to mimic the withdrawal process. Following the induction of withdrawal, a conditioned stimulus (CS) associated with the withdrawal experience is presented alongside the opportunity to self-administer the opioid. The CS may be a sensory cue, environmental context, or other cues that have been previously paired with opioid administration or withdrawal. The presence of the CS during the self-administration session serves to suppress the animals’ motivation to engage in drug-seeking behavior. The suppression of operant responding is then measured by comparing the rate of lever pressing or other operant responses during CS presentations to baseline response rates in the absence of the CS. This suppression of operant responding reflects the aversive effects of withdrawal and the impact of withdrawal-related cues on drug-seeking behavior (Schulteis et al., 2000).

## 4 Extended amygdala overview

In the preceding sections, we have outlined the relevance of negative affective experiences and emotional dysregulation in the phenomenon of opiate withdrawal and prolonged abstinence. We also described how allostatic principles can be used to explain the progressive nature of the loss of control feature of OUD. These behavioral, cognitive and affective adaptations necessarily supervene upon discrete neuroplastic changes, particularly within what is known as the extended amygdala. In this section, we provide more detail about the roles of these component regions of the extended amygdala, including an overview of their constituent neurons and the unique plasticity mechanisms that pertain to them from opioid withdrawal.

The amygdala is a brain region found in the medial temporal lobe that is anterior to the hippocampus. It is comprised of several nuclei that mediate affective, autonomic, motivational and endocrine functions, associative learning and addiction-related processes. The concept of the extended amygdala, proposed by Alheid and Heimer (1988); Heimer and Alheid (1991), is supported by numerous neuroanatomical studies which highlight the structural symmetry, cytoarchitectural, and functional similarities between its component regions. These subregions include the bed nucleus of the stria terminalis (BNST), central nucleus of the amygdala (CeA), and a transition zone within the nucleus accumbens (NAc) shell. Each subdivision of the central and medial amygdala has a corresponding subdivision in the lateral and medial BNST yielding interconnected and continuous columns. Additionally, subregions of the extended amygdala share similar inputs and outputs from with other brain regions, such as the hypothalamic and brainstem regions and the ventral tegmental area (VTA), periaqueductal gray (PAG) area, basolateral amygdala (BLA), hippocampus, perirhinal and entorhinal cortices (McDonald et al., 1999; Baidoo and Leri, 2022).

### 4.1 Substructures and constituent neurons

The extended amygdala comprises a network of brain regions enriched with neuropeptides that includes the CeA, BNST, and the NAc shell and we will describe inputs, outputs and functioning of each (Heimer and Alheid, 1991; Koob, 2020).

#### 4.1.1 Central nucleus of the amygdala

The CeA is situated at the central core of the amygdala and receives executive, contextual, sensory and visceral inputs from a host of regions (Tillman et al., 2018) including sensory inputs coming from the central portion and the midline nuclear complex of the thalamus as the termination region of the thalamo-amygdaloid projections. Hypothalamic inputs also come from ventromedial hypothalamus and from other nuclei that include paraventricular and arcuate nuclei and the lateral hypothalamus (Aggleton et al., 1980); these inputs facilitate homeostatic and somatic functions. Brainstem inputs provide somatosensory, taste, vestibular, and auditory information via the PAG, the pars compacta of the substantia nigra, the VTA, and nuclei of the medulla (e.g., dorsal raphe nucleus, lateral parabrachial nucleus, solitary tract nuclei, interpeduncular nucleus). Somatosensory inputs are also provided from the ventrolateral nucleus of the spinal cord (Aggleton et al., 1980; Norita and Kawamura, 1980). In addition, the CeA and BLA each receive rich cholinergic innervation and the major source of input into the amygdala is from afferents originating from the nucleus basalis of Meynert (Emson and Lindvall, 1979).

Contextual information is processed from various hippocampal subregions including the CA1 region, entorhinal cortex, the subiculum and the perirhinal cortex, medial part of the temporal pole, insula, and parahippocampal regions. These regions provide contextual and cue information to the CeA which attaches emotional valence these stimuli. Inputs from a variety of frontal, associative and temporal cortical regions project mostly to the BLA which serves as an important hub for sensory and executive information processing to the CeA (Rosene and Van Hoesen, 1977; Di Marino et al., 2016).

The CeA has extensive outputs, including connections to the striatum, hippocampus, and neocortex, as well as projections to the hypothalamus, thalamus, brainstem, and basal brain regions. Efferent fibers provide outputs through ventral or dorsal pathways. The ventral amygdalofugal pathway provides a linkage for motivational and emotional functions, through the limbic system, and can influence behavioral responses (Di Marino et al., 2016). This ventral pathway targets the structures of septal region, the sublenticular region (including the basal nucleus of Meynert), the BNST, the thalamus (Price and Amaral, 1981) and the lateral hypothalamic area (Nauta, 1969). Other ventral amygdalofugal fibers provide outputs to various levels of the brainstem and spinal cord and impact stress and motor responses (Mizuno et al., 1985). Dorsal outputs from the CeA also project to septal nuclei, diagonal band nucleus, NAc and hypothalamic nuclei. Post-commissural fibers terminate at the BNST, the mediodorsal nucleus of the thalamus and to the posterior hypothalamus which impacts energy balance, blood pressure, memory, and learning (Di Marino et al., 2016).

CeA efferent projections target the CA1 and CA3 fields of the hippocampus which are involved in the formation, consolidation, and retrieval of hippocampal-dependent memories (Amaral and Cowan, 1980). However, most of the primary projections from the amygdala to the hippocampus originate in the BLA and medio-basal nuclei. It is the BLA and basomedial nuclei that also project towards all the cortical areas of vision, the temporal, frontal and occipital areas and influence sensory experience. The CeA is connected to the BNST via two major fiber bundles—the ventral amygdalofugal pathway and the stria terminalis (Price and Amaral, 1981). The CeA and the BNST both anatomically and functionally activate key features of fear and anxiety via projections to the brainstem, midbrain, and hypothalamic nuclei There is structural symmetry and cytoarchitectural, and functional similarities between the CeA and the BNST. Each subdivision of the central and medial CeA has a corresponding subdivision in the lateral and medial BNST (Di Marino et al., 2016; Krabbe et al., 2018; Tillman et al., 2018).

Striatal projections from the CeA influence both the somatomotor system and the locomotor system, resulting in locomotion and mobility changes (Holstege, 1991), which are part of the “fight or flight” response seen in anxiety and opiate withdrawal. Other motor responses associated with these connections produce opiate withdrawal-induced somatic symptoms, such as vocal emotional expressions, head rotations, urinary dyscontrol, and anxious facial reactions and tics The amygdalar outputs to the hypothalamus (Price and Amaral, 1981) results in major endocrinological impacts including regulation hormonal activity such as the release of adrenocorticotropic hormone (ACTH) and corticotrophin-releasing factor (CRF) in response to stress and drug withdrawal and can be responsible for allostasis. The CeA can influence both sympathetic and parasympathetic effectors, resulting in physiological responses such as increased heart rate, elevated blood pressure, dry mouth, dilated pupils, paleness, sweating, and piloerection. Moreover, hyperactivity of the CeA can contribute to gastrointestinal functional disorders and colonic crises in some individuals, also contributing to allostatic load (Di Marino et al., 2016; Tillman et al., 2018). *These connections enable the CeA to mediate expression of fear and stress responses and provide a crucial link between sensory processing and behavioral and physiological outcomes.* Indeed, because of its role in processing inputs related to chronic anxiety-like states, and its role in stress, induction of the CeA is thought to be critical to the development of allostatic load (Schulkin et al., 1994).

#### 4.1.2 Bed nucleus of the stria terminalis

The BNST is a basal brain structure that borders on edge of the caudate nucleus and the septal region of the amygdaloid body while passing through the stria terminalis. As mentioned, the BNST and CeA demonstrate anatomical and structural symmetry and similar projections to the brainstem, midbrain, and hypothalamic nuclei which activate key signs of fear and anxiety. The BNST receives many cortical inputs from the limbic lobe, insula, the subiculum, and the entorhinal cortex (McDonald et al., 1999). It receives major amygdaloid inputs from the lateral and BLA nuclei (Petrovich and Swanson, 1997). It also receives hypothalamic inputs from the ventromedial and paraventricular nuclei. It also receives inputs from brainstem centers from the PAG, VTA, bulbar nuclei and parabrachial, solitary and dorsal nuclei of the vagus nerve (Sofroniew, 1983; Di Marino et al., 2016). Accordingly, the BNST receives emotional, somatosensory, motivational and autonomic inputs.

The BNST provides efferent connections to the hypothalamus (dorsomedial, ventromedial, paraventricular, supramammillary nuclei and lateral hypothalamus), the thalamus (thalamic nuclei of the midline) and various levels of the brainstem. Efferent projections also target the dopaminergic neurons of the pars compacta of the substantia nigra and VTA, the PAG (autonomic function, motivation, and behavioral responses to threat), and serotonergic neurons of the raphe nuclei (modulating anxiety). BNST neurons also project to midbrain nuclei of the solitary tract (autonomic functions), the noradrenergic locus coeruleus (LC) neurons, the dorsal nucleus of the vagus nerve, and parabrachial nuclei and to the reticular formation (arousal functions) (Dong and Swanson, 2006a; 2006b; Di Marino et al., 2016).

There is a major influence of NE on BNST neurons, and the BNST receives major noradrenergic inputs from the nucleus of the solitary tract and plays a role in dopamine-norepinephrine neurotransmitter interactions through connections with the VTA, substantia nigra and LC. This interplay with noradrenergic and dopaminergic systems affects aversion and anxiety and motivation, respectively, and can greatly influence negative affective phenomena in withdrawal (Di Marino et al., 2016) through its putative role in the development of allostatic load (Elman and Borsook, 2018; Feliu-Soler et al., 2020).

#### 4.1.3 Nucleus accumbens shell

The NAc is a heterogeneous area composed of both core and shell regions, delineated by anatomical connectivity. The NAc core subregion interacts with brain regions associated with motor circuitry and behavioral outputs, while the NAc shell interacts with limbic and autonomic brain regions and plays a key role in reward, aversion, and other emotional and visceral responses to stimuli (Everitt et al., 1999). Within each subregion, the principal medium-spiny neurons (MSNs) are GABAergic neurons that are the primary target of excitatory glutamate afferents and dopamine neurons. These MSNs are divided into two types activated by different dopamine receptors, type 1 (D1) and type 2 dopamine receptors (D2) (Le Moine and Bloch, 1995).

The NAc shell, a subregion of the brain’s reward system, plays a critical role in regulating motivation, reward-related behaviors, and stress and anxiety responses. It is anatomically and functionally distinct from the NAc core. The NAc shell receives inputs from various cortical and subcortical structures, including the BLA, hippocampus, hypothalamus, brainstem, and visceral- and olfactory-related cortices (Brog et al., 1993). These inputs are involved in processing both rewarding and aversive information, potentially contributing to the aversive properties of opiate withdrawal and thus contribute to negative allosteric load. The NAc shell projects to specific targets, notably the ventromedial part of the ventral pallidum which impact motor responses. The NAc shell sends efferents to thalamic mediodorsal nucleus and has strong reciprocal connections with the prelimbic and insular cortex, which are involved in aversion-related behaviors (Groenewegen et al., 1993; Zahm, 1999).

There are differences in the neuroanatomy of the NAc shell and NAc core. Morphological differences are found in dopamine-containing axons and axon terminals in the core that are distinct from those in the shell. Afferents of the shell are largely derived from cortical and subcortical structures that are segregated from those projecting to the core (Alheid and Heimer, 1988). Efferent connections from the NAc shell selectively innervate subregions of the ventral pallidum which projects to the thalamic mediodorsal nucleus. The NAc shell also innervates distinct subregions of the hypothalamus (Zahm et al., 1999). The connectivity of the medial shell with subcortical and brainstem structures combined with these neurochemical distinctions suggest that the NAc shell represents an extension of the extended amygdala and bridges basal ganglia and centromedial amygdaloid patterns of neural organization (Zahm, 1999).

The NAc shell is thought to be functionally relevant to the emergence of allostasis largely through its role in the facilitation of rewarding and aversive experiences. In the brain’s attempt to maintain physiologic homeostasis, activation of the reward system (process (a)) triggers the corresponding activation of the anti-reward system, including the stress system (process (b)). In addition, it is hypothesized that chronic exposure to highly rewarding stimuli (e.g., substances with high potential for addictive involvement), however, leads to chronically high levels of extracellular dopamine, which is offset by a downregulation of dopamine receptors within the NAc, resulting in hypodopaminergia and associated anhedonia (Koob, 2013; Thompson et al., 2021). This anhedonia overlaps with the hyperkatifeia (negative reinforcement and the negative emotions of addiction) that accompanies the chronically upregulated stress system characteristic of allostatic overload (Koob, 2020).

#### 4.1.4 Basolateral nucleus of the amygdala

Though the BLA is not considered as a subregion of the extended amygdala it serves as a critical center for sensory information processing to the CeA and is a region included in this review because of its large significance in mediating opiate withdrawal. The BLA is a cortical-like structure comprising the lateral, basal, and basomedial or accessory basal nuclei, all of which consist of both glutamatergic principal cells and GABAergic interneurons. The flow of information between the BLA and the CeA is mostly unidirectional and linked by multiple parallel pathways involving several types of GABAergic cells. The BLA receives inputs from the thalamus and sensory cortices, enabling the integration of sensory information relevant to conditioned stimuli (McDonald et al., 1999). The BLA plays a pivotal role in relaying sensory inputs to the CeA and maintains reciprocal connections with other key regions, such as the PFC, which provides drug cue information, as well as the ventral hippocampus, which contributes information about drug context (Krettek and Price, 1978; Capogna, 2014). Thus, the BLA acts as a major source of sensory inputs into these interconnected regions and mediates the processing of drug-related information and the generation of behavioral responses.

*Conceptually, the BLA is considered as the main input station of the amygdala for conditioned stimulus information while the CeA is viewed as the main output station for conditioned fear responses which are particularly relevant to allostasis during opiate withdrawal*. BLA neurons play a key role in responding to conditioned stimuli and via projections to the CeA nucleus and then to brainstem and hypothalamic fear effectors. The fear response pathways include CeA neurons projecting to brainstem fear effectors that are primarily contained within its medial section. The lateral amygdala projects to the lateral CeA, which in turn sends GABAergic neurons to the medial CeA, creating a feedback loop. It is hypothesized that fear expression and fear extinction cells in the BLA form a variety of connections within the basal amygdala, as well as with GABAergic inputs to intercalated neurons and medial CeA neurons (Pare and Duvarci, 2012).

## 5 Functional relevance of extended amygdaloid subregions to opiate withdrawal

In this section, we discuss the relevance of each subregion within the extended amygdala to the facilitation of acute and prolonged opioid withdrawal, before describing their respective various neuroplasticity mechanisms impacting this process.

### 5.1 Role of the central nucleus of the amygdala in opioid withdrawal

As described, the CeA plays a vital role in neural circuits that trigger various changes in response to stress, including autonomic, neuroendocrine, and behavioral adjustments, all of which are intrinsic to allostatic-driven adaptations related to opioid withdrawal. The CeA influences hypothalamic CRF systems which act as a central regulator of the hypothalamic-pituitary-adrenal (HPA) axis that coordinates the body’s stress response by stimulating the production and secretion of cortisol from the adrenal cortex. CRF has a broad regulatory influence on multiple organ systems, including behavioral, endocrinological, reproductive, metabolic and autonomic functions and cardiovascular and gastrointestinal activity both at the central and peripheral levels (Slominski, 2009).

Early studies have identified the CeA and other limbic regions as key mediators of the opioid withdrawal response. For instance, in experiments with morphine-dependent rats, administration of intracerebral naloxone to the CeA resulted in withdrawal-induced jumping behavior. Moreover, bilateral electrolytic lesions of the CeA successfully eliminated this withdrawal jumping in rats. This seminal study specifically highlighted the CeA’s central role in mediating behavioral effects of morphine withdrawal (Calvino et al., 1979). Subsequent studies using bilateral excitotoxic lesion techniques in rat models further demonstrated that morphine dependence can be modified through lesions in the amygdala, including the CeA and the BLA. In this study conducted by Watanabe et al. (2002), a CPA paradigm was employed to examine the intracerebral effects of naloxone. Lesions in CeA attenuated withdrawal-induced CPA, while lesions in the BLA had minimal impact. These two studies highlighted the significance of the CeA in both somatic and affective aspects of opioid withdrawal.

Findings from these early lesion studies were corroborated by other approaches that identified the specific locations of withdrawal-induced neuronal activity. For example, an imaging study compared local cerebral metabolic rate for glucose in 84 brain regions of rats treated with naloxone and/or morphine, with saline controls. Naloxone alone caused alterations in the local metabolic rate for glucose in several discrete brain regions, including the extended amygdala (Kraus et al., 1996). Notably, the CeA exhibited an increase in metabolic activity in naloxone-treated rats, suggesting a tonic influence of endogenous opioids upon these regions. Co-administration of morphine reversed this metabolic change, indicating the specificity of naloxone’s metabolic actions for opioid receptors. Another approach utilized immunocytochemical localization of neural activity marker c-Fos in opiate withdrawal. Jin and associates (2005) demonstrated neuronal activation using immunohistochemical studies with the IEG c-Fos in the CeA and BLA following naloxone-precipitated withdrawal in morphine dependent rats. CPA was also tested in morphine withdrawing rats using a range of naloxone doses. In rats given a single morphine exposure, naloxone dose-dependently resulted in CPA. In morphine dependent rats, naloxone treatment produced a dose-dependent increase in c-Fos immunoreactivity within the CeA that was associated with CPA in morphine dependent rats (Jin et al., 2005). The findings in the extended amygdala were evaluated by Gracy et al. (2001), who observed that morphine-dependent rats showed increased c-Fos immunoreactivity in the CeA, NAc shell, and BNST, when treated with higher doses of naloxone. Neural c-Fos values correlated with CPA scores in the naloxone-paired chamber among morphine-dependent rats. Consequently, these results provide support for the hypothesis that the CeA plays a role in the aversive effects induced by opioids, as measured by CPA.

The pharmacological inhibition of the CeA has also been shown to effectively block the expression of opioid withdrawal-induced startle behavior. Cabral et al. (2009) investigated the effects of short-term naltrexone-induced withdrawal at 24 and 48 h post-withdrawal on anxiety-related behaviors. Rats undergoing morphine withdrawal were subjected to the startle reflex paradigm to assess anxiety levels during these two time periods. Notably, at 24 h of withdrawal, an enhanced startle response was observed, which was effectively inhibited by intra-CeA injections of the inhibitory GABA-A agonist, muscimol. These findings provide evidence that the CeA plays a crucial role in mediating the potentiation of startle response during withdrawal (Cabral et al., 2009).

The CeA plays a dual role in mediating fear responses and avoidance behaviors associated with stress responses. Administration of a CRF antagonist into the CeA of morphine-dependent rats resulted in the reversal of withdrawal-induced CPA. Moreover, the administration of immune-toxins to CRF neurons in the CeA produced a reduction in cue-induced operant responding in morphine-dependent subjects. These findings emphasize the significance of blocking intra-amygdala CRF systems, underscoring the role of CeA CRF neurons in mediating behavioral and aversive responses during withdrawal (Heinrichs et al., 1995).

Other studies demonstrated the involvement of CRF neurons in the CeA as crucial to mediating opiate withdrawal. Experimental studies using morphine-dependent rats receiving naloxone injections revealed the significance of CRF mRNA in this process (McNally and Akil, 2002). *In situ* hybridization studies measuring CRF mRNA showed that rats undergoing naloxone-precipitated withdrawal had elevated mRNA levels in the CeA, but not in the BNST. Intracerebral injections of CRF-1 antagonist to the CeA effectively mitigated the severity of somatic opiate withdrawal signs. However, antagonist administration to the BNST did not impact withdrawal. These studies underscore the role of CeA CRF neurons and the CRF-1 receptor (CRF-1) in modulating the effects of opioid withdrawal.

Noradrenergic activity in the CeA also plays a crucial role in the generation of aversive and anxiety-related behaviors in opioid withdrawal. As an amygdaloid nucleus, the CeA receives dense projections of dopaminergic, noradrenergic, and adrenergic pathways (Asan, 1998). Watanabe and associates (2003) demonstrated increased NE levels in the CeA using *in vivo* microdialysis during naloxone-precipitated morphine withdrawal in rats. Furthermore, intra-CeA injections of beta-adrenoceptor antagonists reduced morphine withdrawal behaviors. Specifically, microinjection of propranolol attenuated various somatic withdrawal signs, such as rearing, wet-dog shakes, teeth chattering, paw shakes, diarrhea, ptosis, and rhinorrhea. Intracerebral treatment with different beta-1 and beta-2 receptor antagonists into the CeA also diminished withdrawal-induced CPA. These findings highlight the contribution of NE within the CeA to the motivational and somatic effects of naloxone-precipitated morphine withdrawal (Watanabe et al., 2003).

### 5.2 Role of neuroplasticity in the central nucleus of the amygdala in opioid withdrawal

In addition to CRF neurons, there are other types of neurons in the CeA that play a significant role in opiate withdrawal. Electrophysiological recordings in CeA parvalbumin (PV+) interneurons revealed increased excitability during morphine withdrawal (Wang et al., 2016). Action potentials from PV + interneurons were recorded in mice undergoing withdrawal after a 6-day morphine treatment. Membrane excitability and the membrane spike number were increased in PV + interneurons, while PV- interneurons did not display these changes. Moreover, PV + interneurons exhibited a lower threshold for action potential initiation. Morphine withdrawing mice receiving optogenetic inhibition of PV + neurons exhibited behavioral changes indicative of decreased anxiety and aversive behaviors, as well as increased motivation for natural rewards. Specifically, these mice spent more time in the open arms of the elevated plus maze (EPM) test, showed a higher preference for saccharin, and demonstrated lower CPA scores. Conversely, activation of PV + interneurons in the CeA induced negative affective withdrawal behaviors. Additionally, morphine withdrawal led to an increase in CeA CRF mRNA levels which were attenuated by optogenetic inhibition of CeA PV + interneurons. The findings of this study highlight the role of plasticity within CeA PV + interneurons in modulating the negative affective states associated with morphine withdrawal through the expression of CRF.

Changes in glutamatergic alpha-amino-3-hydroxy-5-methyl-4-isoxazolepropionic acid (AMPA) receptor subunits (or GluA) were found to regulate opioid withdrawal-related behaviors (Cai et al., 2020). This study utilized adeno-associated virus (AAV) vectors to achieve GluA1 overexpression (AAV-GluA1) in the CeA. They first demonstrated that bilateral microinjection of AMPA receptor protein into the CeA attenuated CPA behavior induced by naloxone-induced morphine withdrawal. To confirm these findings, the study infused bilateral AAV-GluA1 vectors into the CeA of rats. Rats receiving a control vector exhibited significant CPA behavior during naloxone-precipitated morphine withdrawal, while AAV-GluA1-infused rats showed reductions in aversive CPA behavior. These findings suggest that the neuroadaptive activation of GluA1 receptors in the CeA inhibits the aversive effects of opioid withdrawal (Cai et al., 2020).

The projections from the CeA to dopaminergic neurons in the VTA play a critical role in mediating the negative motivational effects observed in opiate withdrawal. **Downregulation of the DA reward system and upregulation of the CRF brain-stress system have been implicated in the behavioral responses of opiate withdrawal** (Koob and Mason, 2016; Koob and Volkow, 2016; Koob, 2020). These mechanisms appear to involve projections from CRF neurons in the CeA to VTA dopaminergic neurons. Jiang and others (2021) employed neuronal activity-dependent labeling methods to examine the recruitment of neuronal ensembles in the VTA, termed morphine ensembles (Mor-Ens), during initial morphine exposure. These ensembles projected to the NAc and induced dopamine-dependent positive reinforcement, as observed in morphine-induced operant conditioning. Electrophysiology and tracing techniques also revealed connections between CeA CRF neurons and ensembles Mor-Ens in the VTA (named the CRF CeA→VTA network) which exhibited neuroadaptive enhancements during morphine withdrawal. Deletion of CRF-R1 in Mor-Ens ensembles during opiate withdrawal produced anxiolytic (increased time spent in the open arm of the EPM), antidepressant (increased struggling time in the TST) and increased social preference scores. Pharmacological blockade or CRISPR- (Clustered Regularly Interspaced Short Palindromic Repeats of genetic information) repression of CRF-R1 in Mor-Ens ensembles and weakened these inhibitory CRF CeA→VTA inputs. Inhibition of this circuit produced less aversive effects as measured by CPA during opiate withdrawal. This study suggests that the neurons encoding opioid reward are regulated by withdrawal induced increases in CRF CeA→VTA inputs and result in negative withdrawal behaviors (Jiang et al., 2021).

To investigate the neuroadaptive role of the N-methyl-D-aspartate (NMDA) N1 receptor subunit gene in the CeA during opioid withdrawal, genetic deletion studies were performed by injecting a recombinant adeno-associated virus (rAAV) into the CeA of conditional knockout mice (Glass et al., 2008). This procedure resulted in reduced expression of the NR1 gene and its protein in CeA neurons. Bilateral local deletion of the NR1 gene did not affect somatic signs of opiate withdrawal but influenced CPA in withdrawing mice. To assess the impact of NR1 knockdown on dendritic plasticity in the CeA, ultrastructural analysis was performed to examine dendritic spine morphology, including measures of cross-sectional area, surface area, and axis length. No significant differences were found in dendritic spine number in sections from mice undergoing opiate withdrawal. However, conditional deletion of the CeA NMDA NR1 receptor subunit gene inhibited naloxone-induced CPA, while somatic signs remained unaffected, in morphine-dependent mice. In sum, these studies indicate that both CeA NMDA and AMPA receptors and their associated neuroplasticity play a role in the aversive properties of opioid withdrawal. These CeA plasticity changes are summarized in Table 1.

**TABLE 1:** Neuroplasticity in the central nucleus of the amygdala (CeA) and bed nucleus of the stria terminalis (BNST).

Author	Plasticity or intervention	Functional effects	Region of interest	Effects on opioid withdrawal
Wang et al. (2016)	Action potentials from CeA PV + interneurons were recorded in mice undergoing withdrawal. Membrane excitability and the number of membrane spikes were ↑ in PV + interneurons, while PV- interneurons did not display these changes. PV + interneurons also exhibited a ↓ threshold for action potential initiation	Mice in morphine withdrawal that received optogenetic inhibition of CeA PV + neurons exhibited ↓ anxiety and aversive behaviors and ↓ CRF mRNA levels. Mice spent ↓ time in the open arms of the EPM test, a ↓ preference for saccharin, and higher CPA scores. Activation of PV + neurons induced negative affective-like behaviors	CeA	CeA PV + interneurons are more excitable during withdrawal and their activation produces ↑ anxiety, dysphoria, and ↓ motivation for natural rewards
Cai et al. (2020)	Microinjection of AMPA receptor protein into the CeA ↓ CPA behavior induced by naloxone-induced morphine withdrawal. Infusion of bilateral AAV-GluA1 vectors into the CeA of rats produced the same effect	Rats receiving a control vector in CeA exhibited significant CPA behavior during naloxone-precipitated morphine withdrawal, while AAV-GluA1- and AMPA receptor infusion ↓ CPA	CeA	Neuroadaptive activation of GluA1 receptors in the CeA inhibits the aversive effects of opioid withdrawal
Jiang et al. (2021)	CeA CRF neurons and morphine ensembles (Mor-Ens) in the VTA (CRH CeA→VTA) exhibited neuroadaptive changes following escalating morphine doses. Changes in this circuit mediate negative behaviors during opiate withdrawal	Deletion of this CRF CeA→VTA circuit during withdrawal resulted in ↑ time spent in the open arm of the EPM and struggle time in the TST	CeA	Escalating morphine treatment produces neuroadaptive changes in the CRF CeA→VTA circuit. This circuit mediates anxiety and behavioral despair-like behaviors in opioid withdrawal
Jiang et al. (2021) (continued)	CRISPR-mediated repression of CRF-R1 in neuronal ensembles in the VTA weakened the CRF CeA→VTA inputs	Repression of this circuity counteracted negative behavioral effects, such as CPA during opiate withdrawal	CeA	Repression of CRF CeA→VTA inputs ↓ withdrawal induced dysphoria
Glass et al. (2008)	Conditional deletion of the CeA NMDA NR1 receptor subunit gene did not affect dendritic spine number	Deletion of NR1 receptor gene ↓ naloxone-induced CPA, while somatic signs remained unaffected in morphine-dependent mice	CeA	Deletion of CeA NR1 gene ↑ withdrawal induced dysphoria
Shalev et al. (2001)	Footshock stress-induced reinstatement of heroin seeking is dependent on length of heroin withdrawal period. This reinstatement is maximal responding after 6 and 12 days of withdrawal from heroin and was effective up to 66 days after withdrawal	Footshock ↑ CRF mRNA levels in the CeA and BNST of heroin-trained rats	CeA and BNST	Footshock stress-induced reinstatement of heroin seeking is maximal between 1–2 weeks of withdrawal and continues for 3 months. Footshock stress ↑ CRF systems in the CeA and BNST and could account for enduring SA during withdrawal
Dumont and Williams (2004)	NA ↑ GABA-A IPSCs in the vlBNST in morphine withdrawn rats. Alpha-1 antagonist (prazosin) and beta-adrenergic antagonist (propranolol) blocked noradrenaline-induced ↑ in vlBNST GABA-A IPSCs	vlBNST sends excitatory projections to VTA and GABA-A IPSCs may inhibit this VTA projection. In withdrawal, NA-induced ↑ in vlBNST GABAergic interneurons may ↓ excitatory drive to VTA	BNST	BNST plasticity contributes to the mesolimbic DA-mediated amotivational state characteristic of opiate withdrawal. This mechanism may account for some effects of propranolol and prazosin on withdrawal
Francesconi et al. (2009)	In long access heroin SA rats, there were impairments of the field potential (LTP) in the juxtacapsular BNST 1–2 weeks after heroin withdrawal	Neuroadaptive reductions in LTP in the jcBNST was associated with an elevation in heroin self-administration during withdrawal	BNST	BNST LTP plasticity may contribute heroin SA and relapse during withdrawal
Luster et al. (2020)	Naloxone-induced withdrawal in mice ↓ amplitude of mIPSC currents in BNST neurons and ↑ mIPSC frequency. Repeated morphine withdrawal induced sex-related bidirectional plasticity in spontaneous IPSCs	Repeated morphine withdrawal induced bidirectional plasticity in spontaneous IPSCs on BNST neurons and ↑ GABA release in male mice and ↓ GABA release in female mice	BNST	Neuroplasticity in the CeA ➔ BNST circuit may contribute sex-related differences in the affective component of withdrawal

### 5.3 Role of the bed nucleus of the stria terminalis in opioid withdrawal

Because of its noradrenergic inputs to the brainstem, the BNST of the extended amygdala is especially relevant to opioid withdrawal. Criner and investigators (2007) examined the role of the BNST, and other regions within the extended amygdala, in the expression of opiate antagonist-induced suppression of operant responding during opioid withdrawal. Rats were trained to lever press for food and were then administered single or repeated doses of morphine. Previous research from the same group had indicated that repeated withdrawal inductions could lead to conditioned withdrawal responses, resulting in suppressed food lever pressing following opiate antagonist administration. Rats in this study received multiple doses of the opioid antagonist methylnaloxonium that were infused into various brain regions after the repeated morphine treatments. The researchers found that methylnaloxonium was more effective in suppressing food responding when infused into the NAc (17.9-fold potency shift), BNST (6.8-fold), and CeA (5.5-fold), compared to non-specific i.c.v. administration, highlighting the significance of all three regions. Overall, the study suggests that the BNST, CeA and NAc mediate the suppression of operant responding during morphine withdrawal.

Noradrenergic cells within the BNST were also demonstrated to have a role in opiate withdrawal (Fuentealba et al., 2000). Extracellular levels of NE in the ventral BNST (vBNST) of saline- and chronic morphine withdrawing rats were studied using *in vivo* microdialysis in rats. Additionally, the tissue concentration of NE was studied at different rostrocaudal levels of the vBNST. Chronic morphine treatment resulted in increased extracellular levels of NE in vBNST. Two days after naloxone-induced morphine withdrawal, there were further increases in the extracellular levels of NE in the vBNST. The presence of UK 14304, an alpha-2 adrenergic agonist, induced a decrease in NE extracellular levels in all experimental groups. The results also showed that the vBNST presents a rostrocaudal gradient of NE and contains nearly 10% of total brain NE. The increase in extracellular NE levels in vBNST induced by chronic morphine treatment and the further increase in NE levels 48 h after naloxone-induced morphine withdrawal suggest that NE within the vBNST is involved in the effects of both morphine dependence and withdrawal.

Other studies support these findings of BNST noradrenergic mechanisms in opioid withdrawal. Fuentealba and workers (2000) also used *in vivo* microdialysis studies to demonstrate naloxone-induced withdrawal increases extracellular levels of NE in the BNST. This study also demonstrated a rostrocaudal gradient of NE concentrations in the BNST. Delfs and associates (2000) utilized a retrograde tract-tracer injected into the BNST to identify noradrenergic afferents during opiate withdrawal. They also labeled noradrenergic neurons with tyrosine hydroxylase and assessed these regions using c-Fos-related activity markers. Their findings revealed that tyrosine hydroxylase-immunoreactive neurons in the A1 area of the caudal ventrolateral medulla, as well as A2 cell groups in the caudal medulla sent projections to the BNST. Comparatively fewer noradrenergic neurons from the pontine locus coeruleus (LC) projected to the BNST. All these afferents displayed activation, as indicated by c-Fos expression following opiate withdrawal. Ascending axons of the medullary A1 and A2 noradrenergic neurons constitute the ventral noradrenergic bundle, while neurons from the pontine locus coeruleus form the dorsal noradrenergic bundle. Lesions of the ventral bundle reduced withdrawal-induced CPA but not somatic signs of withdrawal. Conversely, lesions of the dorsal bundle or the LC did not impact aversive or somatic withdrawal signs of withdrawal. Microinjections of beta-1 and beta-2 noradrenergic antagonists into the BNST diminished opiate withdrawal-induced CPA. The alpha-2 adrenergic agonist clonidine, commonly used to alleviate somatic symptoms of opiate withdrawal in humans, also produced a reduction in withdrawal-induced CPA when injected into the BNST. This study highlighted the critical involvement of noradrenergic projections from the caudal medulla to the BNST in the aversive effects of opiate withdrawal (Delfs et al., 2000).

Cecchi and others (2007) examined the roles of the BNST in the somatic and motivational components of opioid withdrawal in rats with high reactivity (HR) *versus* low reactivity (LR) to novelty. During the initial day of spontaneous morphine withdrawal, HR rats displayed increased teeth chattering and eye twitching as compared to LR rats. This study examined adrenergic receptor gene expression in the BNST and found that HR rats showed a selective increase in beta-1 adrenoreceptor expression. To explore the functional relevance of this difference, they microinjected betaxolol, a selective beta-1 receptor antagonist, into the dorsal BNST. The administration of this antagonist dose-dependently decreased teeth chattering during withdrawal in HR rats and blocked opiate withdrawal-induced CPA in HR but not LR rats. These findings support the role beta-1 adrenoreceptors in the BNST in somatic and aversive aspects of opiate withdrawal (Cecchi et al., 2007).

Harris and Aston-Jones (2003) examined the role of the BNST in morphine reward following opiate withdrawal. Rats received chronic morphine treatment followed by a 1-month period of spontaneous opiate withdrawal and then were tested in the CPP paradigm. Rodents undergoing morphine withdrawal showed higher morphine preference scores for the drug-paired environment compared to chronically placebo-treated rats. Morphine-pretreated rats, vs. placebo-pretreated rats, displayed greater c-Fos expression in the ventrolateral BNST, but not in the dorsolateral BNST, compared to control rats. In morphine pretreated rats, there was a positive correlation between place preference measures and c-Fos expression in the ventrolateral BNST (vlBNST) (Harris and Aston-Jones, 2003). Harris and Aston-Jones (2007) also investigated motivational changes for natural rewards during prolonged opiate withdrawal. They employed a similar experimental design, using a chronic morphine pellet implantation followed by CPP testing for food rewards. In the CPP device, the animals were alternately placed in one compartment containing cereal or the other compartment with an empty food container. This process was repeated and a place preference test was performed after prolonged abstinence. They study demonstrated induction of c-Fos expression in the vlBNST, CeA, and noradrenergic neurons (A2) in the nucleus tractus solitarius in morphine-withdrawn animals tested for food preference. The number of c-Fos-positive neurons in these areas exhibited a negative correlation with food preference in abstinent animals. These findings suggest reduced hedonic processing for food rewards, but not morphine reward, during prolonged morphine withdrawal in these stress-related brain areas of the extended amygdala along with its medullary noradrenergic inputs (Harris and Aston-Jones, 2007).

Fox and colleagues (2017) examined the hypothesis that DA and NE signaling interact reciprocally during opiate exposure and withdrawal. To investigate this, they utilized voltammetry to measure catecholamine release in rats exposed to morphine and naloxone-precipitated withdrawal. They compared DA transmission in the NAc with NE concentration changes in the vBNST, and correlated neurotransmitter changes with withdrawal-related behaviors. Acute morphine treatment increased DA transients in the NAc but had no effects on NE responses in the vBNST. Conversely, DA output was decreased during opiate withdrawal, while NE was released in the vBNST during specific withdrawal symptoms. Both NE and withdrawal symptoms could be induced by administering naloxone with an alpha-2 adrenoreceptor antagonist in morphine dependent rats. These findings support reciprocal roles for DA and NE neurotransmission during drug exposure and withdrawal. In summary, these studies support a role for the noradrenergic signaling in the BNST in the somatic and motivational behaviors of opiate withdrawal.

### 5.4 Withdrawal-related neuroplasticity in the bed nucleus of the stria terminalis

Multiples studies have demonstrated the presence of neuroplasticity in the BNST during opiate withdrawal. Shalev and associates (2001) showed the effects of stress on the reinstatement of heroin seeking following different withdrawal periods, as well as the involvement of CRF mRNA in the BNST and CeA. Rats were initially trained to self-administer heroin, underwent extinction, and then experienced footshock-induced reinstatement of heroin seeking. The reinstatement of lever-pressing behavior in response to the footshock stimulus did not occur on day 1 of withdrawal, but it progressively increased and reached its peak on days 6 and 12 of heroin withdrawal. Responding was still present on days 25 and 66 of withdrawal. Increases in CRF mRNA levels were found in the dorsal BNST (on days 1 and 6 of withdrawal) in response to footshock-induced reinstatement, as well as in the CeA (on day 1 of withdrawal). It is hypothesized that the dysphoric and somatic symptoms of heroin withdrawal are associated with neuroplastic elevations of CRF mRNA levels in the BNST and CeA, which may contribute to stress-induced reinstatement (Shalev et al., 2001).

A subregion of the BNST, known as the juxtacapsular division of the lateral BNST (jcBNST), was shown to mediate opiate withdrawal behaviors through a distinct neuroplastic mechanism. The jcBNST receives dense glutamatergic projections from the BLA, via stria terminalis, and then projects back to the anterior BLA and to CeA, lateral hypothalamus, striatum, and NAc (Dong et al., 2000). Francesconi and associates (2009) demonstrated that a form of long-term potentiation (LTP) in jcBNST neurons responded to high-frequency stimulation of the stria terminalis. Rats were trained to self-administer heroin on either a short access (i.e., 1 h per day) or a long access schedule (i.e., 23 h per day). In long access heroin SA rats, there were impairments in the field potential in the jcBNST for one-two weeks after heroin withdrawal. Short access rats also exhibited a partial level of impairment after cessation of SA. These effects were associated with an elevation in heroin SA. The study demonstrated that chronic opiate exposure escalated SA during withdrawal and resulted in reductions in long-term potential in the jcBNST, likely impacting its connections with the BLA, CeA, NAc, and other key regions involved in drug reward. This persistent decrease in the firing threshold of jcBNST neurons may contribute to the anxiety and stress-related relapse mediated by the BLA and CeA during opiate withdrawal.

Luster and others (2020) investigated plasticity in GABAergic signaling within the BNST during opiate withdrawal in mice. After 3 days of morphine administration, mice demonstrated a sensitization of withdrawal symptoms. Electrophysiology experiments were conducted in the BNST 1 day after naloxone-precipitated withdrawal. All morphine withdrawing mice exhibited a variety of electrophysiological changes in the BNST. Withdrawal produced a decrease in the amplitude and increase in frequency of miniature inhibitory postsynaptic currents (mIPSCs). Withdrawal elevated the paired-pulse ratio in all mice suggesting a presynaptic mechanism. Morphine withdrawal induced sex-related bidirectional plasticity in spontaneous IPSCs on BNST neurons, increasing GABA release in male mice and decreasing GABA release in female mice. These findings suggest that neuroadaptations in BNST inhibitory signaling and the divergence of plasticity changes in male and female mice following morphine withdrawal (Luster et al., 2020).

Another potential plasticity mechanism contributing to aversive properties of opiate withdrawal involves BNST inhibition of VTA dopamine neurons. Neurons projecting from the VTA to the vlBNST were identified through retrograde transport of fluorescent microspheres injected into the VTA and through whole-cell voltage clamp recordings in vlBNST neurons. Acute morphine withdrawal increased miniature GABA_A_-IPSCs through an adenylate cyclase-protein kinase A pathway. NE increased spontaneous GABA_A_-IPSCs in the vlBNST of morphine withdrawn rats and alpha-1 antagonist (prazosin) and beta-adrenergic receptor blockade (propranolol) blocked noradrenaline-induced increase of GABA_A_-IPSCs. Downstream adenylyl cyclase blockade inhibited NE-induced increases of spontaneous GABA_A_-IPSCs in slices from morphine withdrawing rats. Given that neurons in the vlBNST send excitatory projections to the VTA, NE may diminish the excitatory drive to mesolimbic dopamine cells during opiate withdrawal via these GABAergic changes in plasticity; this effects may contribute to the amotivational state of opiate withdrawal (Dumont and Williams, 2004). These BNST plasticity changes are summarized in Table 1.

### 5.5 Role of the nucleus accumbens shell in opioid withdrawal

Impairments in negative affect processing during opiate withdrawal may arise from circuit dysfunction in the mesolimbic DA pathway, which is believed to mediate anhedonia-like behaviors and reduce motivational approach behaviors (Ploski and Vaidya, 2021). Sensory afferents to the NAc shell receive conditioned sensory information from the BLA, hippocampus, hypothalamus, brainstem, and visceral- and olfactory-related cortices, which can contribute to the aversive features of opiate withdrawal. Efferents from the NAc shell innervate the ventromedial part of the ventral pallidum that projects to thalamic mediodorsal nucleus with strong limbic connections and could mediate aversion-related behaviors (Zahm, 1999). The NAc integrates both dopaminergic and glutamatergic inputs to modulate the rewarding and aversive properties of opiates. Medium-spiny neurons (MSNs) in the NAc shell are divided into two types activated by different DA D1 and D2 receptors (Le Moine and Bloch, 1995).

Given that mesolimbic DA activation plays a crucial role in producing the rewarding effects of various drugs of abuse, it is hypothesized that the suppression of DA output in the limbic forebrain are associated with the aversive symptoms during withdrawal. In line with this hypothesis, Rossetti and associates (1992) demonstrated that withdrawal from chronic ethanol, morphine, cocaine, and amphetamine resulted in a substantial reduction in extracellular dopamine concentration in the ventral striatum, as measured by *in vivo* microdialysis. After naloxone-precipitated morphine withdrawal, the time course of dopamine reduction mirrored that of the withdrawal symptomatology (Rossetti et al., 1992). These findings were complemented by electrophysiological evidence following morphine withdrawal. Diana et al. (1995) demonstrated that 1 day after chronic morphine treatment, the neural activity of mesoaccumbal DA neurons, as indicated by firing rate and burst firing, was significantly reduced compared to chronic saline-treated controls. However, the administration of morphine restored these electrophysiological parameters. Similarly, when rats were tested after an intravenous challenge with the opiate antagonist naloxone, there was a sharp and substantial decrease in dopaminergic firing rate and burst firing rate. These results suggest that the mesolimbic DA system is chronically downregulated during morphine withdrawal syndrome, leading to a depressed extracellular release of dopamine in the NAc. Diana and coworkers (2006) showed that spontaneous and naloxone-induced withdrawal produces an enduring but reversible decrease in NAc shell spine density in shell, as compared with core neurons. This effect persists up to 14 days when spine density was found within pretreatment values. These findings imply that the hypoactivity of DA neurons during withdrawal may be related to the dysphoric state associated with morphine withdrawal and could lead to increased vulnerability to relapse in an effort to restore euthymia.

Enoksson and researchers (2012) conducted a study in transgenic mice to examine the immunofluorescence of the activity-related IEG, c-Fos, and to investigate separate populations of DA D1-and D2-receptor expressing neurons. Chronic morphine-treated mice underwent naloxone-precipitated withdrawal, and the effects on c-Fos expression were examined in the NAc of transgenic mice that selectively expressed the D2 receptor. The study found that c-Fos expression in the NAc core and shell was increased 2 h after naloxone injection during withdrawal. Furthermore, c-Fos immunoreactivity was predominantly observed in D2 receptor-positive neurons. This study also examined naloxone-precipitated withdrawal in transgenic mice that exclusively expressed D1 receptors. The withdrawal-induced increase in c-Fos expression was also mostly confined to presumed D2 receptor-expressing (D1 receptor-negative) neurons. These findings highlight the distinct neuronal responses occurring in the two DA receptor populations of MSNs within the NAc in response to morphine withdrawal (Enoksson et al., 2012).

A study by Radke and Gewirtz (2012) examined the effects of administration of DA receptor agonists on opiate withdrawal. In their study, intra-accumbal administration of the D1-like receptor agonist SKF82958 and the D2-like receptor agonist quinpirole, as well as systemic administration, were tested after morphine withdrawal. Neither agonist decreased the morphine withdrawal-potentiated startle responses. However, when rats instead received a systemic injection of both SKF82958 and quinpirole after morphine withdrawal, there were decreases in startle responses. The investigators proposed that the potentiated startle response during opiate withdrawal is dependent on reduced activity at both D1-and D2-like receptors, and the administration of D1/D2 receptor agonists prevents the expression of opiate withdrawal-potentiated startle by activating both receptor types (Radke and Gewirtz, 2012).

The transcription factor deltaFosB (DFosB) has been shown to be induced by drugs of abuse highly rewarding substances and activities in the NAc. Nunez and others (2010) investigated the expression of FosB transcription factor plus DFosB proteins in brain stress and motivational systems during morphine withdrawal. Rats were made dependent on morphine and then injected with either saline or naloxone to induce withdrawal. Naloxone-induced withdrawal resulted in expected somatic behaviors and increases in systemic ACTH and corticosterone levels, indicating stress responses. Acute withdrawal led to increases in immunoreactivity of FosB/DFosB in various brain regions, including the extended amygdalar regions of the NAc shell, CeA, BNST, noradrenergic inputs from the nucleus tractus solitarius, and hypothalamic regions of the paraventricular nucleus. This study highlights the expression of FosB/DFosB in different components of the brain stress and motivational systems during morphine withdrawal (Núñez et al., 2010).

Noe and associates (2019) investigated the functional implications of withdrawal-induced hypodopaminergic activity in the NAc shell using a conditioned suppression paradigm of operant food seeking. Rats were trained to lever press for food pellets and then were chronically treated with morphine or vehicle. During naloxone-precipitated withdrawal, the conditioned cue was presented in the operant cage context, resulting in the suppression of lever pressing. In a subsequent cue-only session, reactivation of the aversive memory associated with opiate withdrawal inhibited lever pressing. Neural analyses revealed the activation of NAc shell, BLA, and hippocampal neurons during withdrawal-induced context and cue exposures, while NAc core neurons responded only during the cue period. These findings suggest the important role of the NAc shell-BLA-hippocampal circuit in suppressing food responding during opiate withdrawal.

### 5.6 Withdrawal-related neuroplasticity in the nucleus accumbens shell

As previously mentioned, neuroadaptive morphological changes in accumbal neurons with DA hypoactivity were demonstrated during opiate withdrawal (Rossetti et al., 1992; Diana et al., 1995). Spiga and colleagues (2005) used the Golgi-Cox staining technique and confocal microscopy to measure MSNs in the NAc after opiate withdrawal. Both spontaneous and naloxone-induced withdrawal resulted in a selective reduction of spine density in NAc shell neurons, while spine density counts in rats chronically treated with morphine were not altered. These findings support the hypodopaminergic hypothesis of opiate withdrawal. Furthermore, the reduction in spine density in NAc shell medium spiny neurons was replicated and persisted for up to 2 weeks before returning to pre-treatment levels.

In a study by Kasture and others (2009), the effects of treatment with a neuroprotective agent, withania somnifera extract, were investigated in rats undergoing chronic morphine treatment and subsequent withdrawal. Golgi-Cox staining and confocal microscopy were used to analyze the brains of rats subjected to spontaneous or pharmacologically precipitated withdrawal. The administration of withania somnifera extract during chronic morphine treatment reduced somatic withdrawal behaviors during withdrawal. Additionally, treatment with the extract prevented the reduction in spine density of the NAc shell observed during spontaneous and naltrexone-induced morphine withdrawal. These results suggest that pretreatment with the neuroprotective agent protects against neuroadaptive reductions in dendritic plasticity and somatic symptoms induced by morphine withdrawal.

Wu and colleagues (2013) examined the excitability of MSNs in the NAc shell during morphine withdrawal through *in vitro* whole-cell recordings. NAc shell MSNs were categorized according to their firing patterns as following: repetitive spike discharge with and without firing cessation (types I and II, respectively), and non-repetitive spike discharge (type III). Findings were that morphine withdrawal (for a period of 10 days) increased the intrinsic excitability in type II MSNs and led to rapid spike adaptation that terminated repetitive spike discharge during the depolarization of type I MSNs. Additionally, the afterhyperpolarization currents in NAc shell MSNs were attenuated after chronic morphine withdrawal. These findings indicate that individual MSNs of the NAc shell exhibit unique electrophysiological properties and undergo neuroadaptation during morphine withdrawal (Wu et al., 2013).

Wu and associates (2012) also demonstrated that withdrawal from repeated morphine exposure led to potentiation of both glutamatergic synaptic strength and intrinsic excitability within the NAc shell. Chronic morphine withdrawal potentiated glutamatergic synapses and increased the probability of glutamate release at synapses of MSNs. This potentiation was characterized by the increased frequency of the miniature excitatory postsynaptic currents (mEPSCs), a decrease in the paired-pulse ratio, and an increase in the ratio of AMPA receptor/NMDA receptor mediated currents. MSN intrinsic excitability was also potentiated through the inhibition of sustained potassium currents via extrasynaptic NMDA receptor activation. Chronic morphine withdrawal produced the downregulation of presynaptic group II metabotropic glutamate receptors (mGluR2/3), whereas mGluR2/3 receptor agonist pretreatment blocked this change. These findings underscore the role of glutamatergic neuroadaptations within the MSN of the NAc shell during chronic morphine withdrawal (Wu et al., 2012).

Russell et al. (2016b) demonstrated the contribution of AMPA receptors within the NAc shell to somatic and affective signs of opiate withdrawal. This study demonstrated the effects of NBQX (2,3-dioxo-6-nitro-7-sulfamoyl-benzo [f]quinoxaline), which is an antagonist of the AMPA receptor, on withdrawal behaviors. Intracerebral administration NBQX into the NAc shell prevented naloxone-induced CPA but not somatic signs in morphine-dependent rats. In morphine-dependent rats, cross-linking of NAc tissue resulted in increased surface to intracellular ratios of GluA1, which returned to control levels after naloxone administration. Fractionation of NAc tissue from naloxone-treated morphine-dependent rats showed a decrease in GluA1 subunits in synaptosomal membranes, indicating compensatory removal of GluA1 from synaptic zones during morphine withdrawal. These changes in the synaptic availability of GluA1-containing AMPA receptors in the NAc shell may contribute to the development of negative affective states in response to naloxone. Similarly, Edwards et al. (2009) examined neuroadaptations in AMPA GluA1 phosphorylation in multiple limbic brain regions during opiate withdrawal. Protein kinase A-mediated GluA1 phosphorylation was increased in the NAc shell, CeA, BLA, VTA, hippocampal CA1 and CA3 subregions, and premotor cortex during spontaneous withdrawal from chronic heroin SA. These two studies highlight the involvement of AMPA receptor neuroadaptations in the NAc shell and other brain regions during opiate withdrawal (Edwards et al., 2009; Russell et al., 2016b).

Re-exposure to environments associated with morphine elicits morphine-seeking behavior even after a prolonged period of withdrawal in rats (Kong et al., 2014). Following operant training for morphine-induced nose-poking, rats underwent different durations of spontaneous withdrawal (1, 10, or 30 days) before being returned to the operant chambers for context-induced SA testing. SA behavior was observed to resume after 1 and 10 days of withdrawal. To investigate molecular mechanisms underlying this behavior, the expression and phosphorylation of GluA1 at serine 845 and serine 831 were examined in the NAc and CeA of rats after 1 or 10 days of withdrawal. The levels of phosphorylation of GluA1 at serine 845 phosphorylation in the NAc were correlated with the intensity of active responses after 10 days of withdrawal. Rats exhibiting stronger motivation for morphine SA following 10 days of withdrawal displayed greater neuroadaptations in GluA1 phosphorylation in the NAc. These findings suggest that alterations in GluA1 phosphorylation in the NAc may contribute to the expression of morphine-seeking behavior during prolonged withdrawal periods.

Madayag and others (2019) investigated glutamatergic transmission in D1-or D2-neurons in the NAc shell and core after repeated morphine treatment. Glutamatergic mEPSCs were measured in D1-and D2-neurons in the NAc core and shell subregions 24 h after repeated morphine injection and 2 h after behavior assessment. The results showed an increase in the frequency of miniature EPSCs (mEPSCs) specifically in D2-MSNs in the NAc shell. In the NAc core, repeated morphine treatment had no effect on the amplitude or frequency of mEPSCs in either D1-or D2-neurons. Furthermore, following chronic morphine treatment and subsequent abstinence, a single re-exposure to morphine triggered a rapid and sustained endocytosis of GluA2-containing AMPA receptors in D1-neurons in the NAc shell. This effect was blocked by the infusion of an AMPA receptor blocking peptide administered into the NAc shell. These findings highlight the role of glutamatergic mEPSCs on D1-and D2-neurons in the NAc shell, via AMPA receptor changes, that are associated with morphine withdrawal (Madayag et al., 2019).

Lefevre and associates (2023) examined the intrinsic excitability and synaptic plasticity of DA D1-or D2-neurons using *ex vivo* electrophysiology. Mice received continuous infusion of morphine over 6 days. Additionally, they received twice-daily injections of saline or naloxone to create continuous or interrupted morphine administration conditions. In the NAc shell, spontaneous excitatory postsynaptic current (sEPSC) amplitudes were higher in D1-MSNs of mice treated with discontinuous morphine compared to those with continuous morphine treatment. A similar trend was observed in D2-MSNs. The study also investigated the effects of continuous and interrupted morphine exposure on excitatory synaptic strength by measuring the ratio of AMPA and NMDA receptor-mediated currents. In male mice only, the AMPAR/NMDAR ratio decreased in D1-MSNs in both morphine groups but not in D2-MSNs. The investigators further examined inhibitory inputs to D1-and D2-MSNs and found that continuous morphine administration increased inhibitory signaling selectively onto D1-MSNs, while interrupted morphine decreased inhibitory input selectively onto D2-MSNs. Discontinuous morphine, as found in withdrawal, increased sEPSC amplitudes in D1-and D2-MSNs and D1-MSN functional outputs. Continuous morphine treatment, which produces opioid dependence induces adaptations that reduced the output of D1-MSNs, which are known to promote reward-related behavior and this may contribute to the amotivational state of dependence/withdrawal (Lefevre et al., 2023).

In a study by Qu and associates (2019), the role of small conductance calcium-activated potassium channels (SK channels) was examined in the NAc shell and medial prefrontal cortex of rats after morphine withdrawal. Rats underwent repeated morphine treatment that yielded CPP and were sacrificed 3 weeks after the final CPP test during a morphine abstinence period. Brain slices were analyzed using *ex vivo* electrophysiology, revealing enhanced spike firing of NAc shell neurons in the morphine abstinence group. Voltage clamp methods were employed to isolate SK currents and morphine abstinence produced a reduction in SK currents in NAc shell neurons. Immunohistochemistry was used to show decreased expression of SK2 and SK3 subunits in the NAc after morphine abstinence. These findings suggest that neuroadaptive changes in small conductance calcium-activated potassium channels in the NAc shell, resulting in enhanced MSN excitability, and play a role in morphine withdrawal and indicate their potential as a target for preventing relapse (Qu et al., 2019). These NAc shell plasticity findings are summarized in Table 2.

**TABLE 2:** Neuroplasticity in the nucleus accumbens (NAc) shell.

Author	Plasticity or intervention and results	Functional or behavioral effects	Region of interest	Importance in opioid withdrawal
Rosetti et al. (1992)	Withdrawal from chronic morphine ↓ extracellular DA concentration in the ventral striatum as measured by *in vivo* microdialysis	The time course of naloxone-precipitated morphine withdrawal behaviors followed the time course of hypodopaminergic neuronal output	NAc	Opiate withdrawal produces hypodopaminergic effects associated with somatic symptoms
Diana et al. (1995)	Spontaneous opiate withdrawal ↓ neural activity of mesoaccumbens DA neurons, as indicated by firing rate and burst firing. Naloxone treatment produced an even greater decrease in DA firing and burst firing rate	Electrophysiological finding findings support the hypodopaminergic hypothesis of opiate withdrawal	NAc	Morphine withdrawal produces ↓ firing in DA neurons following the time course of withdrawal which produce dysphoria
Spiga et al., 2005 Diana et al. (2006)	Spontaneous and naloxone-induced withdrawal resulted in a reduction in spine density in NAc shell neurons (Spiga) Reductions in spine density in NAc shell MSNs persisted for up to 2 weeks of withdrawal before returning to pre-treatment levels (Diana)	Opiate withdrawal produces reductions in NAc shell structural plasticity that follows the behavioral time course of withdrawal	NAc shell	Opiate withdrawal produces ↓ in NAc extracellular DA ↓ DA cell firing and reductions in NAc structural plasticity that follow time course of withdrawal
Kasture et al., 2009	Reduction in spine density in the NAc shell observed during spontaneous and naltrexone-induced morphine withdrawal	Neuroprotective agent injections given during repeated morphine treatment ↓ somatic withdrawal behaviors and prevented the reduction in spine density	NAc shell	Opiate withdrawal ↓ NAc shell plasticity associated with somatic symptoms that was reversed by a neuroprotective agent
Wu et al. (2013)	Prolonged morphine abstinence after chronic treatment ↑ intrinsic excitability and spike adaptation in a subtype of MSNs. Afterhyperpolarization currents were ↓ after withdrawal	MSNs in the show unique electrophysiological properties which might contribute to morphine withdrawal-induced neuroadaptation	NAc shell	Morphine withdrawal ↑ MSN excitability changes; type-specific electrophysiological functions are required to understand withdrawal effects
Wu et al. (2012)	Chronic morphine withdrawal potentiated glutamatergic synapses and ↑ the probability of glutamate release in MSN synapses	Morphine withdrawal potentiates glutamatergic synaptic strength via ↑ frequency of mEPSCs, ↓ paired-pulse ratio and ↑ ratio AMPAR/NMDA receptor-mediated currents	NAc shell	Morphine withdrawal produces ↑ excitability of glutamatergic currents in the NAc
Wu et al., 2012 (continued)	Chronic morphine withdrawal ↑ MSN excitability via inhibition of potassium currents via extrasynaptic NMDA receptor activation. Withdrawal produced downregulation of presynaptic group II metabotropic glutamate receptors (mGluR2/3) and enhanced glutamate release probability	Withdrawal from repeated morphine exposure potentiates glutamatergic synaptic strength and intrinsic excitability in the NAc shell. Pretreatment with an mGluR2/3 agonist prevented the potentiation, indicating the involvement of mGluR2/3 receptors	NAc shell	Morphine withdrawal potentiates MSN neuronal excitability and is treatable with metabotropic mGluR2/3 receptor agonist which could reduce relapse potential during withdrawal
Russell et al., 2016a	In morphine-dependent rats, cross-linking of NAc tissue resulted in ↑ surface to intracellular ratios of GluA1 which returned to control levels. Fractionation of NAc tissue from withdrawing rats showed a decrease in AMPA GluA1 subunit in synaptosomal membranes	Administration of the AMPAR antagonist NBQX into the NAc shell prevented naloxone-induced CPA in morphine-dependent rats	NAc shell	Morphine withdrawal involves AMPA GluA1 plasticity in the NAc shell. Treatment with AMPA receptor antagonist blocks the aversive effects of opiate withdrawal
Edwards et al. (2009)	Spontaneous withdrawal from chronic heroin SA ↑ protein kinase A-mediated GluR1 phosphorylation in the NAc shell and other regions	Findings highlight the involvement of AMPA receptor neuroadaptations in the NAc shell during opiate withdrawal	NAc shell and other extended amygdala regions	Mechanism of phosphorylation of GluA1 receptors may mediate NAc shell neuronal changes in opioid withdrawal
Kong et al. (2014)	Following morphine SA, rats underwent spontaneous withdrawal. SA behavior returned after 1 and 10 days of withdrawal. Expression and phosphorylation of GluR1 at serine 845 (Ser845) and serine 831 (Ser831) were examined	The level of GluR1-Ser845 phosphorylation in the NAc correlated with the intensity of active SA responses after 10 days of withdrawal	NAc	Alterations in GluA1 phosphorylation in the NAc may contribute to the expression of morphine-seeking behavior during prolonged withdrawal periods
Madayag et al. (2019)	Chronic morphine treatment ↑ in the frequency of mEPSCs in D2-MSNs in the NAc shell but not core. During subsequent abstinence, a single re-exposure to morphine triggered a rapid and sustained endocytosis of GluA2-containing AMPARs in D1-MSNs	AMPA receptor blocking peptide administered into the NAc shell reversed endocytosis of GluA2 receptors in D1-MSNs	NAc shell	Treatable neuroplastic AMPA receptor changes occurring in D1- and D2-neurons in the NAc shell are associated with morphine withdrawal
Lefevre et al., 2023	Mice received either continuous or discontinuous morphine treatment over several days. The amplitude of sEPSCs was higher in D1- and D2-MSNs of mice treated with discontinuous morphine. Continuous morphine ↑ inhibitory signaling selectively onto D1-MSNs, while interrupted morphine ↓ inhibitory input selectively onto D2-MSNs	Continuous morphine administration produced neuroadaptations that ↓ the output of D1-MSNs which are known to mediate reward	NAc shell	Continuous morphine which produces opioid dependence ↓ D1-MSN functional outputs and which may produce blunted reward effects and amotivational states
Qu et al., 2019	Rats underwent repeated morphine via CPP paradigm and then underwent morphine abstinence. Brain slices from abstinent mice showed enhanced spike firing in NAc shell neurons. Morphine abstinence ↓ small conductance calcium-activated potassium (SK) currents in NAc shell neurons. SK2 and SK3 subunits in the NAc were ↓ after morphine abstinence	Neuroadaptive changes in small conductance calcium-activated potassium channels play a role in enhanced spike firing in the NAc during morphine withdrawal	NAc shell	Plasticity changes in small conductance calcium-activated potassium channels may be potential targets for treatment during opiate withdrawal

### 5.7 Role of the basolateral nucleus of the amygdala in opioid withdrawal

The BLA serves as the major conduit for sensory information processing to the CeA and has large impacts in opiate withdrawal. It receives and processes cortical, thalamic and hippocampal sensory information related to drug and withdrawal and has efferent connections to the medial CeA which projects to the hypothalamus, midbrain, LC, and PAG which serve as effectors for withdrawal behaviors. Roozendaal et al. (2002) demonstrated the presence of CRF receptors in the BLA that are critical to stress-induced memory consolidation. Martínez-Laorden and associates (2020) utilized the CPA paradigm to evaluate the role of BLA CRF/CRF1 receptor signaling on corticosterone plasma levels. In this study, opiate dependent mice demonstrated somatic withdrawal symptoms. They then underwent a CPA conditioning paradigm with naloxone and demonstrated place aversion along with increases in plasma corticosterone. Intracerebral CRF1 antagonist treatment in the BLA reversed withdrawal CPA demonstrating that withdrawal is a potentially treatable stress-related phenomena.

García-Pérez and Milanés (2020) conducted a CPA study examining the roles of NA and DA neurotransmission within the BLA during opiate withdrawal. The analysis of BLA punches revealed reductions in NE, its metabolites, and NE turnover during morphine withdrawal CPA testing. Conversely, morphine withdrawal CPA led to increases in DA metabolites, specifically dihydroxyphenylacetic acid, and DA turnover in the BLA. Furthermore, a negative correlation was observed between NA turnover in the BLA and body weight loss during withdrawal. A positive correlation was found between BLA DA and dihydroxyphenylacetic acid/DA turnover, and the CPA score in morphine withdrawn rats. These results indicate that the BLA NA system is associated with somatic symptoms, while the BLA DA system modulates the negative affective state of withdrawal (García-Pérez and Milanés, 2020).

Wu and others (2014) conducted a study utilizing the both CPP and CPA paradigms to investigate the role of the BLA and its adrenoreceptors in the reconsolidation of morphine-associated emotional memory in rats. The experiment involved conditioning morphine withdrawing rats to a specific context. After contextual reactivation, the researchers infused a protein inhibitor, anisomycin, into the BLA, which disrupted the reconsolidation of CPP at 4 days, 1 week, and 2 weeks after withdrawal. In other experiments, the beta-adrenoreceptor receptor propranolol was bilaterally administered to the BLA and resulted in impaired reconsolidation of CPA. These findings indicate that aversive memories associated with opiate withdrawal undergo reconsolidation within the BLA, requiring protein synthesis and beta-receptor adrenergic transmission (Wu et al., 2014).

GABA is another important transmitter in the BLA that plays a role in opiate withdrawal behaviors. Seno and coworkers (2022) examined the role of intra-BLA administration of GABA-A agonist and GAD inhibitor in startle potentiation and freezing behavior produced by morphine-precipitated withdrawal. Intra-BLA treatment with GABA-A agonist reduced naloxone withdrawal-induced freezing behaviors while GAD inhibitor inhibited the amplitude of the contextual fear-potentiated startle during withdrawal. Researchers hypothesized that increases in BLA GABAergic transmission may interfere with or inhibit the formation or storage of fear and drug memories.

### 5.8 Neuroplasticity in the basolateral nucleus of the amygdala in opioid withdrawal

Frenois and colleagues (2002) localized the neural circuit involving the BLA responsible for the somatic and motivational effects of opiate withdrawal. In morphine-dependent rats, different doses of naloxone induced various somatic signs of withdrawal. The lower naloxone dose primarily produced some somatic signs, while the higher dose led to additional systemic signs like diarrhea, weight loss, and jumping behaviors. Motivational signs of withdrawal, demonstrated through naloxone-induced CPA, were also observed at multiple naloxone doses, triggering c-Fos mRNA expression in the BLA, lateral septal nucleus, and CA1 regions of the hippocampus. Higher naloxone doses activated DA structures (VTA and striatum), the NA region of the LC, hypothalamic nuclei, and the PAG. Interestingly when the number of c-Fos-expressing neurons increased in the CeA it conversely decreased in the BLA. *Their hypothesis was that inhibitory BLA GABA interneurons control the activity of BLA excitatory glutamatergic efferent projections to the CeA.* Naloxone-induced c-Fos mRNA expression in the CeA was hypothesized to result from the loss of inhibitory control over glutamatergic efferents in the BLA.

In a translational model of heroin SA and withdrawal, Hellemans et al. (2006) the role of the BLA in suppressing opioid seeking was examined. Rats were implanted with bilateral cannulae in the BLA and trained to self-administer heroin by responding on a drug seeking lever in an operant paradigm. Rats were then made opiate dependent via continuous infusion of heroin using mini-osmotic pumps and were subsequently conditioned to associate a stimulus (CS) with naloxone, which triggered CPA. The presence of the heroin withdrawal led to inhibition of heroin-seeking, possibly due to impairments resulting from withdrawal. Furthermore, the IEG Zif268 protein was upregulated in the BLA, but not in the CeA, 2 h after re-exposure to the withdrawal-conditioned stimulus. When antisense oligonucleotides targeting Zif268 were administered into the BLA, there was an impairment in the CS-mediated suppression of heroin seeking. These findings identify the role of the BLA in suppressing opioid seeking during opiate withdrawal and suggest that drug memories undergo protein synthesis-dependent reconsolidation in this region (Hellemans et al., 2006).

Frenois et al. (2005) conducted a study to identify the specific types of neurons in the BLA and CeA involved in opioid withdrawal. The BLA primarily consists of glutamate output neurons (GAD-) with a smaller population of GABA interneurons expressing glutamate decarboxylase (GAD+), the enzyme involved in GABA synthesis. In contrast, the CeA primarily contains GAD + neurons. By performing double-labeling of GAD and IEG c-Fos in the BLA and CeA, the study revealed that acute opiate withdrawal reduced the density of c-fos/GAD + neurons (GABAergic neurons) in the BLA and increased their density in the CeA. Then re-exposure to withdrawal-related conditioned stimuli increased the density of these neurons in the BLA and reduced their density in the CeA, indicating a neuroplastic response. These findings suggest that withdrawal memories produce activity-dependent neuroplastic and reciprocal changes in different subpopulations of amygdalar neurons (BLA and CeA) involved in aversive conditions such as opiate withdrawal.

A variety of studies highlight the importance of DA pathways from the BLA in opiate withdrawal. Lintas and coworkers (2011) examined the intra-BLA effects of DA D1 and D2 antagonist on morphine CPP in heroin withdrawing mice. In opiate-naive rats, blockade of intra-BLA D1, but not D2, receptor transmission blocked the acquisition of opiate CPP. In opiate dependent and withdrawal-conditioned rats, intra-BLA D2, but not D1, receptor blockade blocked opiate reward indicating a neuroadaptive switch in mechanisms. Single-unit *in vivo* extracellular recordings were performed NAc neurons in the opiate-dependent/withdrawn state and showed increases in dopaminergic firing frequency. This suggests an important BLA-NAc shell projection relevant to opiate dependence and withdrawal.

Lyons and coworkers (2013) demonstrated the ability of intra-BLA DA D1 or D2 receptor transmission modulate the motivational salience of opiates via a D1-mediated extracellular signal-regulated kinase- (ERK) dependent mechanism in the opiate-naive state. They found that in the state of opiate dependence/withdrawal, BLA systems switch from a D1-ERK mechanism to a D2-mediated calcium/calmodulin-dependent protein kinase (CaMKIIα) mechanism. Opiate dependence/withdrawal also produced a downregulation of ERK1/2 phosphorylation and a reduction in both total and phosphorylated CaMKIIα signaling in the BLA. In sum, opiate exposure state changes functional control of BLA ERK1/2-dependent *versus* CaMKIIα-dependent memory mechanisms.

Rosen and coworkers (2017) demonstrated that chronic heroin reduces the expression of the DA D3 receptor in the BLA. They showed intra-BLA DA D3 receptor blockade in the BLA with had no effect on opiate reward memory formation in opiate naïve animals. In opiate dependent rats, intra-BLA DA D3 receptor antagonist treatment prevented the formation of opiate reward and withdrawal aversion memory. In summary, there are important BLA dopaminergic signaling pathways involving DA D1, D2 and D3 pathways and downstream DA effectors of ERK 1/2 and CaMKII that play key roles in opiate dependence and withdrawal. There are neuroadaptive switches in these mechanism in naïve rodents that undergo morphine dependence/withdrawal.

Song and coworkers (2019) examined the importance of projections between the PrL cortex and the BLA in the conditioned retrieval of opiate withdrawal memories. To identify this projection, FluoroGold was injected into the PrL to retrogradely label BLA neurons. Morphine dependent mice were trained in a naloxone-induced CPA paradigm. To inhibit this BLA-PrL projection and impact opioid withdrawal-induced CPA, optogenetic silencing was employed. When the optical fiber was activated above the PrL, the projection was inhibited and resulted in the elimination of withdrawal-induced CPA. Optogenetic activation of the BLA inputs to PrL neurons and subsequent feedback signals produced a plasticity-related process in other BLA neurons which were not identified as projection neurons. This study also demonstrated a feedback circuit from the PrL to the BLA using a two-step virus injection approach. BLA neurons projecting to the PrL play an important role in conditioned context–induced retrieval of withdrawal memories and PrL activation induces a neuroplasticity–related feedback process in BLA neurons relevant to these memories.

Shao and associates (2021) conducted a study examining the role of the BLA-anterior cingulate cortex (ACC) projection in the formation of early morphine withdrawal memories and for the longer term transfer of this information to the ACC. Fluorogold was injected into the ACC to label projection neurons. A chemogenetic approach was used to influence this projection in a rodent model of morphine withdrawal-induced CPA. Inhibition of BLA–ACC projection neurons had no effects on the retrieval of long-term CPA withdrawal memory. But repeated chemogenetic inactivation of this BLA-ACC projection did block CPA and resulted in its blockade 14 days later. This produced the inhibition of long-term withdrawal memory formation and decreased in IEG expression in the ACC. The findings suggested that the persistent activation of the BLA-ACC projection following the formation of early withdrawal memory plays a crucial role in the transfer of memories to the cerebral cortex to form long-term memories via plasticity of ACC neurons.

Deji and associates (2022) discovered that another projection originating from the BLA to the ventral hippocampus plays a key role in mediating anxiety during opiate withdrawal. Using a mouse model, they employed anterograde and retrograde viruses to track the activation of this projection, along with c-Fos to measure activity in both regions. During the first 2 weeks after morphine withdrawal, mice exhibited anxiety-like behaviors, as assessed by the EPM and OFT. Optogenetic and chemogenetic inhibition of BLA inputs to the ventral hippocampus produced reduction in anxiety-like behaviors in mice in morphine withdrawal. Furthermore, knockdown of the kappa opiate receptor in the BLA-ventral hippocampus circuit not only diminished its function but it also alleviated anxiety-like behaviors and prevented stress-induced CPP reinstatement. These findings underscore the importance of the BLA-ventral hippocampus projection in modulating anxiety-like behaviors during opiate withdrawal and highlight the kappa opioid receptor as a target for therapeutic interventions.

Yuan et al. (2020) utilized a rat model of heroin SA, consisting of several days of SA followed by extinction training, in order to examine the role of the BLA in heroin seeking. After 1 month of SA training, the rats were exposed to either a noncontingent heroin injection (UCS), or a drug-associated sound (CS), 1 h before extinction sessions. Exposure to the UCS, but not CS memory, prior to extinction training reduced priming-induced reinstatement of heroin seeking. The combination of chemogenetic reactivation of BLA and CS exposure during extinction procedures also inhibited extinction retrieval. Chemogenetic inactivation of the BLA eliminated the inhibitory effects of UCS on extinction procedures and increased heroin seeking. Chemogenetic activation of the BLA restored the inhibitory effects of the UCS on extinction retrieval and reduced heroin seeking. AMPA GluA2 receptor trafficking in the BLA was required for the inhibitory effect of UCS extinction manipulation on heroin seeking. These findings emphasize the involvement of the BLA in the reconsolidation of heroin-associated memory after prolonged withdrawal that involves AMPA receptor trafficking (Yuan et al., 2020).

Protein kinase M ζ (PKMζ) has an important role in neuroplasticity as it increases the number of active postsynaptic AMPA receptors, enhances excitatory synaptic transmission, and has a role in LTP. In a study conducted by He and colleagues (2011), the effects of a PKMζ inhibitor in the BLA on aversive and reward memories was examined during morphine withdrawal in rats. Subjects undergoing morphine withdrawal were tested for the expression of CPA and the following day and received injections of either the PKMζ inhibitor or vehicle into the BLA or the CeA. The study demonstrated intra-BLA injection of the PKMζ inhibitor impaired the expression of CPA on the day following injection, while intra-CeA injection had no effect. In another group, PKMζ inhibitor was microinjected into the BLA 1 day after testing for morphine CPP. The protein kinase inhibitor blocked the expression of CPP on days 1, 7, and 14 of retesting. These findings indicate that PKMζ in the BLA is necessary for the maintenance of associative morphine withdrawal-associated aversion and reward memories (He et al., 2011). These BLA plasticity changes are summarized in Table 3.

**TABLE 3:** Neuroplasticity in the basolateral nucleus of the amygdala (BLA).

Author	Plasticity or intervention and results	Functional or behavioral effects	Region of interest	Importance in opioid withdrawal
Frenois et al. (2002)	Naloxone induced withdrawal produced dose-dependent ↑ in c-Fos expression in the BLA.	Naloxone produced ↑ somatic signs, while the higher dose and ↑ motivational effects of CPA associated with BLA c-Fos expression	BLA and other regions (CeA, lateral septal nucleus, hippocampus)	Expression of c-Fos in the CeA and the BLA during withdrawal produced variation in opposite directions. Inhibitory BLA GABA interneurons control the activity of BLA glutamatergic efferent projections to the CeA
Hellemans et al. (2006)	Rats trained to SA heroin were made opiate dependent via chronic administration of heroin followed by withdrawal. Withdrawal-related CPA led to inhibition of heroin SA. SA produced IEG Zif268 upregulation in the BLA	Administration of antisense Zif268 oligonucleotides produced impairment in the CS-mediated suppression of heroin seeking	BLA	The BLA plays a role in suppressing opioid seeking during opiate withdrawal and drug memories undergo protein synthesis-dependent reconsolidation
Frenois et al. (2005)	Performance of double-labeling of GAD (GABA marker) and the activation marker c-Fos mRNA in the BLA and CeA showed that acute opiate withdrawal ↓ the density of c-Fos/GAD + neurons (GABAergic neurons) in the BLA and ↑ their density in the CeA	Re-exposure to withdrawal-related CS ↑ the density of GABAergic neurons in the BLA and ↓ their density in the CeA, indicating reciprocal and neuroplastic response	BLA and CeA	Opiate withdrawal memories produce activity-dependent and reciprocal neuroplastic changes in the BLA and CeA involved in the aversive conditions of opiate withdrawal
Lintas et al. (2011)	In opiate-naive rats, blockade of intra-BLA D1, but not D2, receptor transmission ↓ opiate CPP acquisition. In opiate dependent/withdrawn rats, intra-BLA D2, but not D1, receptor blockade ↓ opiate reward via an adenylyl cyclase mechanism	Single-unit *in vivo* extracellular recordings performed in neurons of the NAc shell in the opiate-dependent/withdrawn state showed neuroadaptive ↑ in D1- and D2- neuronal firing frequency	BLA and NAc Shell	Findings suggest plasticity in important BLA-NAc shell projections relevant to opiate dependence and withdrawal and indicating a switch in dopamine receptor mechanisms
Lyons et al. (2013)	The ability of BLA DA D1 or D2 receptor transmission to modulate the motivational salience of opiates. It operates through a D1-mediated ERK-dependent mechanism in the opiate-naive state, but switches to a D2-mediated CaMKIIα-dependent mechanism in the dependent/withdrawn state	Opiate dependence and withdrawal produce downregulation of ERK1/2 phosphorylation and a reduction in both total and phosphorylated CaMKIIα signaling	BLA	Opiate exposure state produces functional control of BLA ERK1/2-dependent *versus* CaMKIIα-dependent memory mechanisms. Opiate withdrawal produces a switch in signaling mechanisms
Rosen et al., 2017	Chronic heroin ↓ expression of the DA D3 receptor in the BLA. Study showed that intra-BLA DA D3 receptor blockade had no effect on opiate reward memory formation in opiate naïve animals	In opiate dependent rats, DA D3 receptor antagonist prevented the formation of opiate reward and withdrawal aversion memories	BLA	There are important BLA D3 signaling pathways involved in opiate dependence and withdrawal. Opiate withdrawal produces a switch in DA D3 signaling mechanisms
Song et al. (2019)	FluoroGold is injected to the PrL to retrogradely label BLA neurons. Morphine dependent mice undergo naloxone CPA and it produced Arc activation in the BLA. Optogenetic silencing of the BLA-PrL projection produced the elimination of withdrawal-induced CPA	Optogenetic inhibition of PrL glutamatergic terminals produced ↑ in withdrawal CPA and ↓ Arc activity in the BLA.	BLA and PrL	BLA neurons projecting to the PrL play an important role in retrieval of withdrawal memories and PrL activation induces a neuroplasticity–related feedback process in BLA neurons relevant to these memories
Deji et al. (2022)	Use of anterograde and retrograde viruses to track the activation of BLA-ventral hippocampus projection, along with the c-Fos to measure activation in both regions. During the first 2 weeks after morphine withdrawal, mice exhibited anxiety-like behaviors, as assessed by the EPM and OFT	Optogenetic and chemogenetic inhibition of BLA inputs to the ventral hippocampus, produced reductions in anxiety-like behaviors in mice demonstrating morphine withdrawal. Knockdown of the kappa opiate receptor in this circuit ↓ anxiety-like behaviors and blocked stress-induced CPP reinstatement	BLA and ventral hippocampus	The BLA-ventral hippocampal projection modulates anxiety-like and reward behaviors during opiate withdrawal and highlight its potential as a target for kappa-opioid therapeutic interventions
He et al. (2011)	Morphine-withdrawn rats demonstrated CPA post-withdrawal. Then, rats received injections of the PKMζ inhibitor into the BLA or the CeA. The expression of CPA was retested 1 day after treatment	PKMζ inhibitor impaired the expression of CPA, while intra-CeA injection had no effect. Intra-BLA treatment with PKMζ inhibitor after testing for morphine CPP ↓ reward effects after retesting	BLA and CeA	Protein kinase M ζ (PKMζ) ↑ AMPA receptors and enhances excitatory synaptic transmission. BLA PKMζ has role in morphine withdrawal reward and aversion
Shao et al., 2021	A chemogenetic approach was used to influence BLA-ACC projections in a morphine withdrawal CPA paradigm. Initial inhibition of BLA–ACC projection neurons had no effects on the retrieval of long-term CPA withdrawal memory	Repeated chemogenetic inhibition of the BLA-ACC projection blocked long-term withdrawal memory formation and ↓ IEG expression in the ACC	BLA and ACC	Persistent activation of BLA-ACC projection after the formation of early withdrawal memory blocks the transfer of memories to the ACC to form long-term memories by promoting ↑ plasticity of these ACC neurons
Yuan et al. (2020)	In a model of heroin SA followed extinction training, rats were later exposed to either an unconditioned heroin stimulus (UCS) or a conditioned stimulus (CS) just before the extinction sessions. The UCS inhibited the retrieval of extinction memory and ↑ Fos expression in the BLA whereas the CS had no effect	Chemogenetic inactivation of the BLA eliminated the inhibitory effect of UCS extinction retrieval procedure and ↓ heroin seeking. Chemogenetic activation of the BLA restored the inhibitory effects of the UCS on extinction retrieval via AMPA GluA2 receptor trafficking	BLA	These findings highlight the importance of BLA in the reconsolidation of heroin-associated memory after prolonged withdrawal that involves AMPA receptor trafficking

## 6 Discussion

OUD is often a chronic disorder that produces tremendous morbidity and mortality. It is characterized by uncontrolled and hazardous use of opioids with impaired inhibitory control over involvement with opioids, resulting role dysfunction, a dysphoric withdrawal syndrome upon discontinuation of opioids, and long-term likelihood of relapse despite negative consequences. With prolonged opioid SA an allostatic state develops in which there is chronic deviation of the neural regulatory system from its normal operating level that is maladaptive, depending upon the lessons learned and predictions made by the opioid dependent individual. Opioid withdrawal is associated with neuroplastic changes as manifested by alterations in neuronal electrophysiology and neural connectivity and molecular changes in key brain areas. There is evidence that the underlying neurobiological vulnerability for OUD consists, in part, of opioid-induced structural and functional synaptic adaptations of the brain’s reward and stress systems. Importantly, these neuroadaptive changes to the stress and reward systems are accompanied by negative affective experiences that commonly give rise to relapse, even after prolonged periods of abstinence.

With persistent opioid use, the positive affective effects of opioids and conditioned stimuli mediated by the neural reward system reflexively trigger an anti-reward system that offsets the excessive reward activation. This anti-reward system becomes functionally “upregulated” by persistent opiate exposure while the reward system is “downregulated” to offset persistent mesolimbic dopaminergic activation. In addition, the amygdala-based stress system also becomes sensitized or upregulated after repeat drug exposure and conditioning to aversive cues and contexts associated with negative aspects of opiate withdrawal. The result of this neuroplasticity is a chronically high state of endogenous stress and a dampened or blunted hedonic state during opioid dependence and abstinence. Additionally, the inhibitory control system of the prefrontal cortex is eroded or undermined by the body’s allostatic tendency to achieve self-control through maladaptive self-regulation. Notably, the affective changes that characterize this opioid-induced allostasis are the result of distinctive neuroadaptations, particularly in the extended amygdala. These neuroadaptations result from neural transcriptional and translational processes, varied molecular mechanisms, and electrophysiological changes in neurons and neurocircuits.

The extended amygdala encompasses a network of brain regions that are involved in motivation, stress responses and the regulation of emotions. It includes highly interconnected regions that include the BNST, the CeA and NAc shell. The CeA is located at the central core of the amygdala and receives and integrates sensory inputs coming from the hippocampus, temporal and parahippocampal regions of the cortex and the thalamus, hypothalamus, brainstem. It receives inputs from a variety of frontal, associative and temporal cortical regions via the BLA which integrates sensory information processing to the CeA (Di Marino et al., 2016). The CeA has extensive outputs, including connections to the striatum, hippocampus, and neocortex, as well as projections to the hypothalamus, thalamus, brainstem, and basal brain regions. These projections provide a linkage for autonomic, stress, motivational, emotional functions that and influence behavioral and motoric responses.

Neurophysiological changes in the CeA during opiate withdrawal include increased excitability of neurons during withdrawal associated with anxiety, dysphoria, and amotivational behavioral states (Wang et al., 2016). AMPARs are enriched in the postsynaptic membrane on dendritic spines, are highly dynamic, and play an important role in synaptic plasticity. Changes in AMPA number, composition, phosphorylation state can modify synaptic strength and support cellular forms of learning (Chater and Goda, 2014). There are neuroadaptations of AMPA GluA1 receptor in the CeA and this change is associated with the conditioned aversive effects of opioid withdrawal (Cai et al., 2020). Additionally, glutamatergic plasticity in the CeA involves NMDA receptor mechanisms. Studies show that the CeA NMDA-NR1 receptor contributes to opioid withdrawal-induced aversive effects and highlight the importance of NMDA plasticity in opioid withdrawal.

The CeA is part of an endogenous CRF circuit that mediates neuroendocrine, autonomic, and behavioral changes in response to stress. The CeA contains CRF-expressing neurons that communicate with widespread regions of the neural axis. A CeA-VTA projection exhibits neuroadaptive changes following escalating morphine treatment and mediates dysphoria during withdrawal (Jiang et al., 2021). Indeed, repression of this CeA CRF-VTA projection reduces withdrawal-induced dysphoria. Also, footshock stress in heroin-trained subjects increases CRF levels in the CeA and BNST (Shalev et al., 2001). Perhaps due to the CRF changes, footshock-induced reinstatement of heroin seeking is maintained for over 3 months after withdrawal. In summary, the glutamatergic and CRF-mediated plasticity in the CeA accounts for many of the aversive, dysphoric and effects of opioid withdrawal and associated vulnerability to relapse.

The BNST and CeA demonstrate anatomical and structural symmetry and similar projections to the brainstem, midbrain, and hypothalamic nuclei which activate key signs of fear and anxiety. The BNST receives many cortical limbic inputs and major amygdaloid inputs through the BLA. Importantly, the BNST receives major noradrenergic inputs from the nucleus of the solitary tract and plays a role in DA-NE neurotransmitter interactions through connections with the VTA, SN and LC. This interplay with noradrenergic and dopaminergic systems affects aversion, anxiety and motivation in opiate withdrawal. BNST neurons also project to midbrain nuclei, NA LC neurons, the dorsal nucleus of the vagus nerve, and the reticular formation important in fear responses, arousal and autonomic activity.

Neurophysiological elements of plasticity in the BNST during opiate withdrawal involve several specific subtypes of neurons. Noradrenaline-induced increases of GABA_A_-IPSC interneurons may contribute to aversion during opioid withdrawal (Dumont and Williams, 2004). Medications that treat some symptoms of opiate withdrawal, including alpha-1 antagonist (prazosin) and beta-adrenergic antagonist (propranolol) block noradrenaline-induced increases in vlBNST GABA_A_ IPSCs, highlighting the importance of this form of plasticity. The BNST contains a dense population of GABAergic interneurons and receives GABAergic input largely through projections from the CeA. It is hypothesized that plasticity changes in the CeA ➔ BNST circuit contributes to the affective component of withdrawal (Luster et al., 2020). Neuroadaptations in BNST pre- and post-synaptic GABAergic neurons, and their noradrenergic influences, may contribute to a variety of affective and aversive behaviors in opiate withdrawal.

The NAc shell is a subregion of the brain’s reward system and is anatomically and functionally distinct from the NAc core. The NAc shell receives inputs from various cortical and subcortical structures, including the BLA, hippocampus, hypothalamus, brainstem, and sensory cortices. These inputs are involved in processing both rewarding and aversive information. The NAc shell projects to the ventral pallidum which impact motor and responses and thalamic mediodorsal nucleus with its connections with the prelimbic and insular cortex involved in aversion-related behaviors (Zahm, 1999).

Neuroplastic changes in the NAc shell during opiate withdrawal include decreases in mesolimbic dopaminergic inputs. Withdrawal from chronic morphine reduces extracellular dopamine concentration in the ventral striatum that is associated with inhibition of somatic symptoms (Rossetti et al., 1992). Spontaneous opiate withdrawal reduces neural activity of dopaminergic neurons, as indicated by firing rate and burst firing and this was associated with dysphoria and reduced reward (Diana et al., 1995). The results of this hypodopaminergic effects of opiate withdrawal are manifested in changes the NAc shell structural plasticity. Spiga and others (2005) show that spontaneous and naloxone-induced withdrawal produces a reduction in spine density in NAc shell neurons and these decreases persist for 2 weeks (Diana et al., 2006). Kasture and associates (2009) demonstrated that these neuroplastic changes in the NAc shell follow the behavioral time course of withdrawal and are reversible with treatment of a neuroprotective agent.

Characteristics of these NAc shell electrophysiological adaptations in opiate withdrawal have been further characterized. Prolonged morphine abstinence after chronic treatment increase intrinsic excitability and spike adaptation in MSNs and reduce afterhyperpolarization currents (Wu et al., 2012). Furthermore, morphine withdrawal increases MSN excitability through the following: increased frequency of mEPSCs, and AMPAR/NMDA receptor-mediated currents and inhibition of paired-pulse ratios and potassium currents via extrasynaptic NMDA receptor activation (Russell et al., 2016a). Withdrawal produces downregulation of presynaptic group II metabotropic glutamate receptors (mGluR2/3) and enhanced glutamate release probability, which also increases MSN excitability; this is reversible by metabotropic glutamatergic agonist treatment. Several studies show that changes in neurophysiological plasticity in MSNs are mediated by AMPA receptor regulation. Treatable changes in the synaptic availability of GluA1-containing AMPARs in the NAc shell develop and appear to contribute the development of negative affective states in opiate withdrawal. A mechanism of phosphorylation of GluA1 receptors may mediate NAc shell neuronal changes in opioid withdrawal and contribute to the expression of morphine-seeking behavior during prolonged withdrawal (Edwards et al., 2009; Kong et al., 2014). Chronic morphine treatment increases the frequency of mEPSCs in D2-MSNs in the NAc shell while morphine re-exposures produces endocytosis of GluA2-containing AMPARs in D1-MSNs (Madayag et al., 2019). AMPA receptor changes are found in D1-and D2-neurons including increased amplitudes of EPSCs in D1-and D2-MSNs in morphine withdrawal paradigms (Lefevre et al., 2023). Morphine abstinence reduces small conductance calcium-activated potassium (SK) currents in NAc shell neurons (Qu et al., 2019). These changes all increase the excitability of MSNs in opiate withdrawal. This hypodopaminergic state may reduce the impact of natural rewards and produce dysphoria while the increase in excitability of NAc shell MSNs may increase vulnerability to relapse. These mechanisms underscore the possibility of treatment of withdrawal with mGluR and AMPA receptor agents and drugs which regulate specific potassium channels.

The flow of information from the BLA to the CeA are linked by multiple parallel pathways involving several types of GABAergic cells. The BLA receives inputs from the thalamus and sensory cortices, enabling the integration of sensory information relevant to conditioned stimuli. The BLA maintains reciprocal connections with key regions such as the prefrontal cortex, which provides drug cue information, and the ventral hippocampus, which contributes drug context information. Thus, the BLA acts as a major source of sensory inputs into these interconnected regions and mediates the processing of drug-related information and the generation of behavioral responses (Capogna, 2014).

Conceptually the BLA is considered as the main input station of the amygdala for conditioned stimulus information while the CeA is viewed as the main output station for conditioned fear responses relevant during drug withdrawal. Frenois et al. (2002) showed that inhibitory BLA GABA interneurons control the activity of BLA excitatory glutamatergic efferent projections to the CeA. Withdrawal-related aversive effects lead to inhibition of heroin SA and produces IEG upregulation in the BLA (Hellemans et al., 2006). This suggests drug memories undergo protein synthesis-dependent reconsolidation in the BLA. Acute opiate withdrawal reduces the density of GABAergic neurons in the BLA and reciprocally increases their density in the CeA (Frenois et al., 2005). The BLA has an important role in the reconsolidation of heroin-associated memory after prolonged withdrawal which involves AMPA receptor trafficking (Yuan et al., 2020). These findings suggest that opiate withdrawal memories produce activity-dependent and reciprocal neuroplastic changes in the BLA and CeA and that BLA effects are AMPA receptor-dependent.

Several projections from the BLA appear to have critical roles in opiate withdrawal including those to the PrL. Optogenetic silencing of this BLA-PrL projection eliminated withdrawal-induced aversion (Song et al., 2019). Projections from the BLA to the ACC are also critical in opiate withdrawal. Shao and others (2021) showed that repeated chemogenetic inhibition of the BLA-ACC projection blocked long-term withdrawal memory formation and reduced IEG expression in the ACC. This suggests that persistent activation of BLA-ACC projection after the formation of early withdrawal memory blocks the transfer of memories to the ACC where long-term drug memories are formed. Projections from the BLA to the hippocampus are also critical to opiate withdrawal behaviors. Optogenetic and chemogenetic inhibition of BLA inputs to the ventral hippocampus reduces anxiety-like behaviors in mice undergoing morphine withdrawal (Deji et al., 2022). This BLA-hippocampal projection modulates anxiety-like and reward behaviors during opiate withdrawal and kappa-opioid receptor knockdown in this circuit reduces anxiety-like behaviors and blocks stress-induced CPP reinstatement. BLA projections to hippocampal and cortical regions create drug memories and mediate conditioned aversive and motivational effects of opiate withdrawal.

**FIGURE 1:**
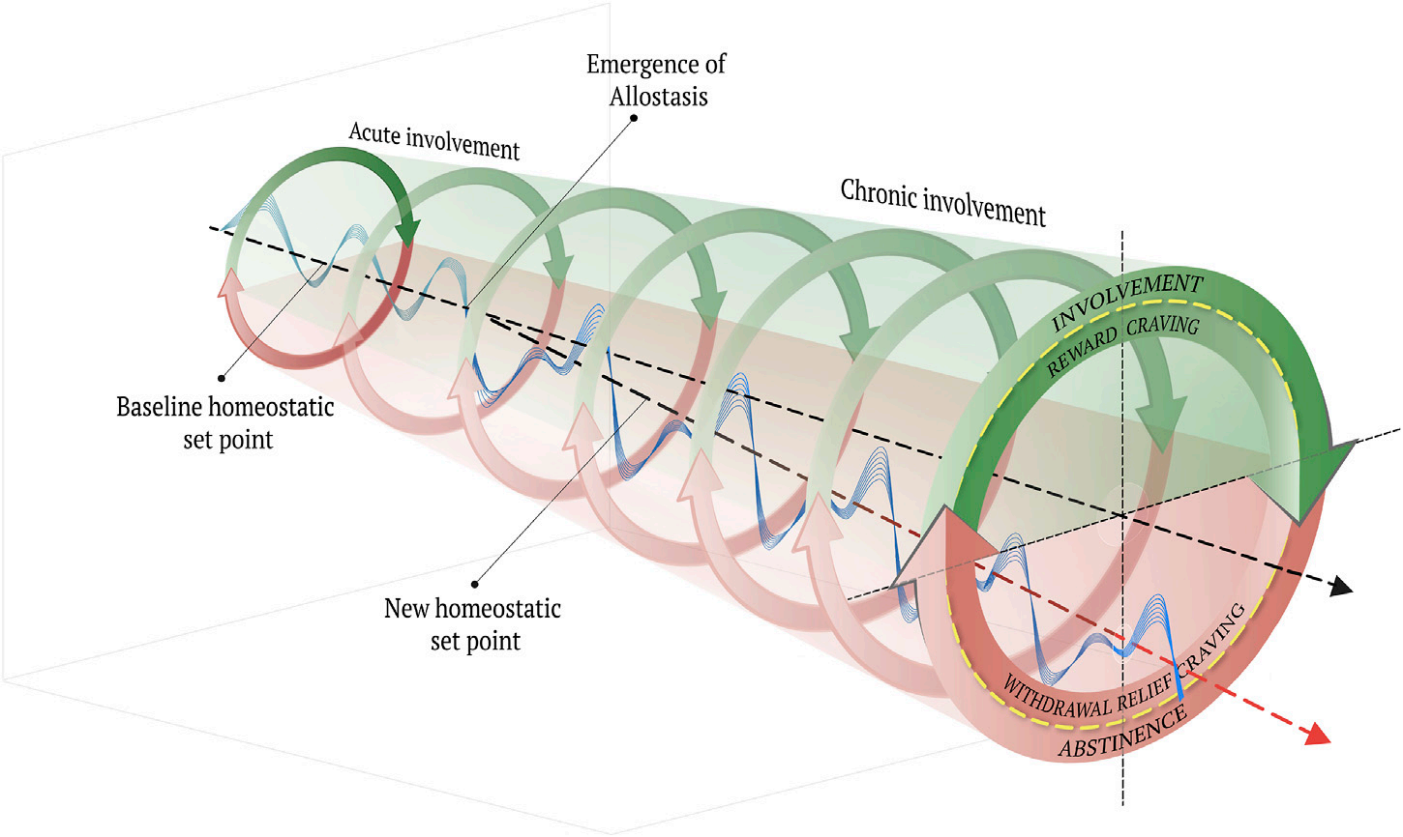
A schematic depiction of the emergence of allostasis through the transition from acute to chronic opioid involvement. The circumplex at the center of the cross-section of this figure represents the affective experiences that correspond to the two discrete behavioral phases of addiction: involvement and abstinence. The recursive arrows of the outer cylinder represent the cyclical nature of the addictive experience, which coincides with both behavior and affective experience. See the text for a summary. The three-dimensional cylinder represents a hypothetical “space” of affective experience through time. The wavefunction represents perpetual departures and fluctuations around the homeostatic set point, which may extend into any of the quadrants (represented from the cross-sectional viewpoint) with variable intensity and frequency, depending on numerous influences for varying durations of time. During acute involvement, there are corresponding affective experiences (i.e., reward craving and withdrawal relief craving), which correspond with a relatively contained range of oscillation of the wave function around the baseline homeostatic set point. Through the process of allostasis, a new homeostatic set point emerges, which, in the case of chronic opioid involvement, tends to be more consistently negatively valenced.

Ultimately, an allostatic framework is integral to understanding the neurobiological mechanisms underlying OUD and opiate withdrawal. Through the process of allostasis, new homeostatic set points develop via neuroplastic changes in key circuits, which are more consistently negatively valenced (Figure 1). The extended amygdala, including the CeA, BNST, and NAc shell, undergoes neuroplastic changes that contribute to the negative affective states, aversion, and relapse behaviors observed in OUD. Furthermore, the BLA’s involvement in processing conditioned stimuli and its projections to various brain regions highlight its crucial role in opiate withdrawal and the allostatic adaptations associated with opioid addiction.

## 7 Conclusion

Despite advances in medication-assisted treatments, OUD remains a widely prevalent condition with tremendous morbidity and mortality. The underlying neurobiological vulnerability for OUD involves structural and functional synaptic adaptations in the brain’s reward and stress systems. Allostasis, the process by which the body achieves stability and adapts to stressors, plays a crucial role in the development and maintenance of OUD. In the context of opioids, allostasis refers to the body’s attempt to achieve stability by predicting future feelings states based on previous opioid involvement and withdrawal. However, this response can lead to neurobiological adaptations that contribute to the chronic negative affective experiences associated with OUD and increase the risk of relapse.

One key aspect of allostasis in OUD is the interaction between the reward system and the stress system in the brain. With prolonged opioid involvement, the positive affective effects of opioids, mediated by the NAc shell, and associated conditioned stimuli trigger an anti-reward system that counteracts the excessive activation of the reward system. This anti-reward system becomes functionally “upregulated” while the reward system is “downregulated” to restore stability. Consequently, individuals with OUD experience a chronically elevated state of endogenous stress and a diminished hedonic state during both opioid dependence and abstinence. Furthermore, opiate withdrawal memories induce activity-dependent and reciprocal neuroplastic changes in both the major input system, via BLA, and the major stress output systems, via the CeA and BNST. These neuroplastic changes contribute to the allostatic process by modulating the transmission of sensory information to the extended amygdala. Consequently, disturbances in the effector output of the CeA and the BNST stress systems may arise, leading to various affective, aversive, and relapse behaviors during opiate withdrawal.

In summary, investigating the intricate neuroplastic changes occurring within the extended amygdala’s reward and stress systems, as part of the allostatic response, provides valuable insights into the mechanisms underlying opioid addiction. By understanding how the brain adapts and maintains stability through allostasis during opiate withdrawal, we can identify potential targets for developing novel treatments that address the negative affective experiences and relapse tendencies associated with this disorder. Opiate dependence and withdrawal reduce neural activity of mesoaccumbens dopaminergic neurons and reduce structural plasticity in the NAc shell, resulting in amotivational effects of opiate withdrawal and abstinence. This hypodopaminergic state produces dysphoria and may reduce the impact of natural rewards while the increase in excitability of NAc shell neurons may increase vulnerability to relapse. Finally, BLA transmission to hippocampal and cortical regions are clearly impactful in the perception of conditioned aversive and anxiety effects of opiate withdrawal by higher executive systems. In conclusion, the prevention or reversal of these many neuroplastic effects in the extended amygdala of opiate dependence or withdrawal could lead to novel and hopeful new interventions in this life-threatening condition.

## Figures and Tables

**Table:** 

**AAV**	adeno-associated virus
**ACC**	anterior cingulate cortex
**AMPA**	alpha-amino-3-hydroxy-5-methyl-4-isoxazolepropionic acid
**BLA**	basolateral nucleus of the amygdala
**BNST**	bed nucleus of the stria terminalis
**CaMKIIα**	calcium/calmodulin-dependent protein kinase IIα
**CB1**	cannabinoid receptor subtype
**CeA**	central nucleus of the amygdala
**CRF**	corticotrophin-releasing factor
**CPA**	conditioned place aversion
**CPP**	conditioned place preference
**CRISPR**	clustered regularly interspaced short palindromic repeats of genetic information
**CS**	conditioned stimulus
**D1**	dopamine receptors, type 1
**D2**	dopamine receptors, type 2
**D3**	dopamine receptors, type 3
**DA**	dopamine
**DFosB**	deltaFosB immediate-early gene
**EPM**	elevated plus maze
**ERK**	extracellular signal-regulated kinase
**GABA**	gamma-aminobutyric acid
**GAD**	glutamatic acid decarboxylase
**GLU**	glutamate
**GluA**	AMPA receptor subunit
**HPA**	hypothalamic-pituitary-adrenal axis
**HR**	high reactivity rats
**IEG**	immediate-early gene
**IPSCs**	inhibitory postsynaptic currents
**jcBNST**	juxtacapsular BNST
**LC**	locus coeruleus
**LR**	low reactivity rats
**LTP**	long-term potentiation
**mEPSCs**	miniature excitatory postsynaptic currents
**mGluR**	metabotropic glutamate receptors
**Mor-Ens**	morphine ensembles
**MSN**	medium-spiny neurons
**NAc**	nucleus accumbens
**NBQX**	2,3-dioxo-6-nitro-7-sulfamoyl-benzo [f]quinoxaline, an AMPA receptor antagonist
**NE**	norepinephrine
**NMDA**	N-methyl-D-aspartate
**NR1**	NMDA receptor subunit
**OFT**	open field test
**OUD**	opioid use disorder
**PAG**	periaqueductal gray
**PKMζ**	protein kinase Mζ
**PrL**	prelimbic cortex
**PVN**	parvalbumin neurons
**rAAV**	recombinant adeno-associated virus
**SA**	self-administration
**sEPSCs**	spontaneous excitatory postsynaptic currents
**SK**	small conductance calcium-activated potassium channels
**TST**	tail suspension test
**UCS**	unconditioned stimulus
**vBNST**	ventral BNST
**VTA**	ventral tegmental area
